# An Episodic Model of Task Switching Effects: Erasing the Homunculus from Memory

**DOI:** 10.5334/joc.97

**Published:** 2020-09-10

**Authors:** James R. Schmidt, Baptist Liefooghe, Jan De Houwer

**Affiliations:** 1 LEAD-CNRS UMR 5022, Université Bourgogne Franche-Comté (UBFC), FR; 2 Department of Experimental Clinical and Health Psychology, Ghent University, BE; 3 Department of Social, Health and Organizational Psychology, Utrecht University, NL

**Keywords:** computational modelling, neural networks, episodic memory, binding, switch costs, feature integration, task-rule congruency, instruction implementation, goals

## Abstract

The Parallel Episodic Processing (PEP) model is a neural network for simulating human performance in speeded response time tasks. It learns with an exemplar-based memory store and it is capable of modelling findings from various subdomains of cognition. In this paper, we show how the PEP model can be designed to follow instructions (e.g., task rules and goals). The extended PEP model is then used to simulate a number of key findings from the task switching domain. These include the switch cost, task-rule congruency effects, response repetition asymmetries, cue repetition benefits, and the full pattern of means from a recent feature integration decomposition of cued task switching (Schmidt & Liefooghe, 2016). We demonstrate that the PEP model fits the participant data well, that the model does not possess the flexibility to match any pattern of results, and that a number of competing task switching models fail to account for key observations that the PEP model produces naturally. Given the parsimony and unique explanatory power of the episodic account presented here, our results suggest that feature-integration biases have a far greater power in explaining task-switching performance than previously assumed.

## Introduction

In our everyday interactions with the world, we often balance multiple goals concurrently, flexibly switching between them as needed. While at work, we might repeatedly shift our attention between checking emails, writing a manuscript, and checking on the Java code running in the background. Understanding how we maintain multiple goals and shift between them, and what costs or benefits might arise from this multitasking, are therefore vital questions that much research has focused on understanding.

**Figure 1:**
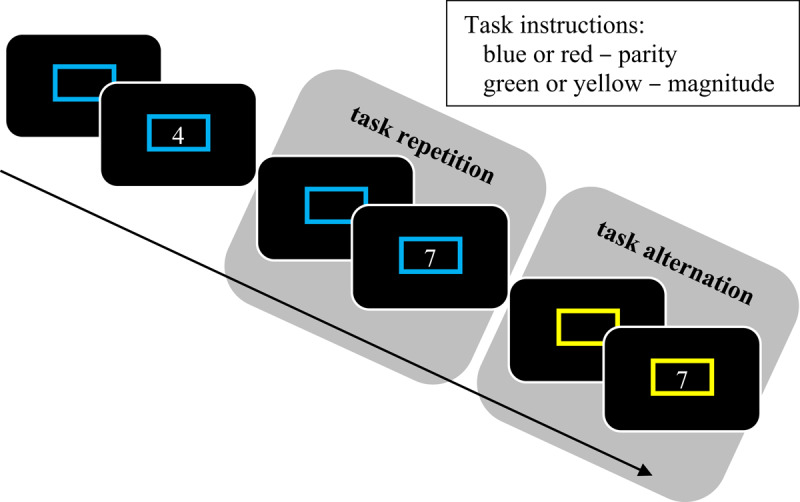
Illustration of a cued task switching procedure. The colour of a cue indicates whether the participant should judge the parity or magnitude of the digit. The task can either repeat or alternate from one trial to the next.

One tool commonly used in the study of our ability to switch between goals are *task switching* paradigms (Jersild, 1927; for reviews, see Kiesel et al., 2010; Koch, Poljac, Müller, & Kiesel, 2018; Monsell, 2003; Vandierendonck, Liefooghe, & Verbruggen, 2010). In task switching paradigms, the participant is not asked to implement one task goal, but two (or more), as illustrated in Figure 1 (for a list of key term definitions, see Appendix A). For instance, in the cued task switching procedure, participants are presented with a cue (e.g., a coloured rectangle) on each trial, which informs them which of two tasks must be performed. For example, a blue or red cue might indicate that participants need to determine whether a target digit is odd or even (parity decision), whereas a yellow or green cue indicates that the participant must determine whether the digit is larger or smaller than five (magnitude decision). The *switch cost* is the observation that performance is substantially hindered when the task on the current trial (e.g., parity) is different from the task on the previous trial (e.g., magnitude), termed a *task alternation* (or *task switch*), relative to when the task is the same, termed a *task repetition*.

There are many different accounts of the origins of switch costs. Most accounts of switch costs postulate the existence of control processes, centered around the notion of *task-sets*. The definition of a “task-set” is certainly open for interpretation, but here we provide the definition from Vandierendonck and colleagues (2010): “a collection of control settings or task parameters that program the system to perform processes such as stimulus identification, response selection, and response execution.” That is, each task-set (e.g., parity and magnitude) promotes a different configuration of the cognitive system for achieving the relevant goal. This is related to the ECTVA theory of Logan and Gordon (2001), where the executive control system sets the “parameters” for visual attention (e.g., which features to attend to), response selection criteria, etc.

According to the *task-set reconfiguration* account, the cost of switching tasks originates from the reconfiguration of the task-set (Meiran, 1996; Rogers & Monsell, 1995). In other words, the task-set can stay as is on a task repetition, but the cognitive system must be “reprogrammed” on a task alternation. According to this account, the switch cost is a rather direct reflection of this reconfiguration time (i.e., to the extent that the reconfiguration is proposed as the sole mechanism contributing to the switch cost). That is, further processing of stimuli must wait on the reconfiguration, which manifests (in a direct way) as the switch cost. Preparatory task-set reconfiguration has also sometimes been linked with the concept of *proactive control* (e.g., Meiran, Kessler, & Adi-Japha, 2008).

Another account, often referred to as the *task-set inertia* or *reactive control account*^1^ (Meiran et al., 2008) assumes that the task-set implemented on the previous trial competes with the task-set that needs to be implemented on the current trial on a task alternation. This competition is thought to occur during execution of the task (Allport, Styles, & Hsieh, 1994). That is, the task-set from the previous trial carries over to the next, which creates a competition on task switches but not on repetitions. This competition is said to delay processing, such that it takes longer for the cognitive system to “settle” on a response, thereby indirectly producing the switch cost. A related notion assumes a facilitative effect of task-set inertia for a repeated task (Yeung & Monsell, 2003a, 2003b). Of course, task-set inertia and reconfiguration are, respectively, dynamic and discretized stage versions of the same idea and need not be viewed as mutually exclusive (i.e., there could be some elements of processing that are dynamic and others that are stage separated; see also Vandierendonck et al., 2010, for a similar point). Accordingly, we use the umbrella-term “task-set control” when referring to these types of accounts in the remainder of the text.

We also note here that task-set reconfiguration and task-set inertia are sometimes additionally/alternatively distinguished by whether the task-set is, respectively, adjusted in a voluntary or involuntary fashion. However, we will focus less on this latter distinction, in part because we interpret the discrete versus dynamic adjustment distinction as more central to these theories and in part because it is much more ambiguous where volition does or does not enter into the types of computational models what we will discuss in the present report. Globally, the reader should note that we discuss these theoretical accounts (along with other terminology) in the way that they are (from our perspective) typically understood, but that alternative conceptualizations remain possible.

In the following, we put forward a very different view on task switching and task-set control to explain several observed results (without denying the important role of cognitive control in executing a task more generally, of course). Our account, which we will implement in an exemplar-based memory framework, assumes that much of what is observed in a task switching experiment (i.e., key effects of interest) is actually due to specific sequences of events that are possible in different conditions. Of particular importance, which combinations of stimuli, responses, cues, etc. that can or cannot repeat from the immediately-preceding trial is not the same for task repetitions and task switches, and this creates systematic biases that inflate the “true” effect of switching from one task to another. Such “feature integration” (or “binding”) effects emerge naturally from an exemplar-based memory framework that relies exclusively on encoding and subsequent retrieval of episodic memories. We aim to show that this simple notion can explain not only the basic switch cost but also a range of findings from the task switching literature, in addition to making some novel predictions that do not follow from competing accounts. We note in advance that our account does share some commonalities with competing accounts (depending on how interpretted; e.g., Gilbert & Shallice, 2002; Schneider & Logan, 2005, 2009; Waszak, Hommel, & Allport, 2003), and will discuss these relations in the General Discussion. We also note in advance that we certainly do not attempt to model everything in one paper (including some important task switching phenomena, which we will return to in the General Discussion, as well).

### Feature integration

It is commonly accepted that the switch cost only partly reflects the difference between a task repetition and a task switch. In particular, the switch cost is heavily biased by *feature integration* (or *binding*) effects (Arrington & Logan, 2004a; Logan & Bundesen, 2003, 2004; Logan & Schneider, 2006b; Monsell & Mizon, 2006; Schmidt & Liefooghe, 2016; Schneider & Logan, 2005), which can inflate the effect attributed to task-set control. Both within and outside the task switching domain, it is generally recognized that repetition of features (e.g., stimulus and response) from one trial to the next can have sizeable effects on performance (Dalrymple-Alford & Budayr, 1966; Hommel, 1998). In task switching procedures, the possible combinations of features that can repeat on a task repetition are completely different than those that can repeat on a task alternation.

For instance, the cue (e.g., blue) can *only* repeat on a task repetition, but never on a task alternation (except with transition cues; see Forstmann, Brass, & Koch, 2007; but see Schneider & Logan, 2007; cf., Van Loy, Liefooghe, & Vandierendonck, 2010). Simply seeing the same cue twice in a row speeds performance considerably (Logan & Bundesen, 2003, 2004; Logan & Schneider, 2006a, 2006b; Logan, Schneider, & Bundesen, 2007; Mayr & Kliegl, 2003; Schneider & Logan, 2005), likely because the repeated cue can be processed more quickly. A substantial proportion of the switch cost (though not all; see Arrington, Logan, & Schneider, 2007; Monsell & Mizon, 2006) is due to faster processing of repeated cues in the task repetition condition and not to a repetition of the task *per se* (for a review, see Jost, De Baene, Koch, & Brass, 2013).

There are yet further feature integration biases of concern (Schmidt & Liefooghe, 2016; Waszak et al., 2003; Waszak, Hommel, & Allport, 2004). For instance, there are also stimulus-response binding confounds in the switch cost. If the stimulus repeats on a task repetition (e.g., “3” followed by “3”), then the (correct) response, by definition, repeats as well (e.g., “3” is always an odd digit, and never even). Performance is exceptionally fast on this type of *complete repetition* trial, because performance can be speeded by retrieval of the just-encoded memory linking the stimulus and response together. In contrast, on a task alternation a repeated stimulus may require a different response. For instance, “7” might require a left keypress to indicate “odd” (parity) on one trial, then another “7” might require a right keypress to indicate “>5” (magnitude) on the next trial (see also, Goschke, 2000; Kleinsorge & Heuer, 1999; Kleinsorge, Heuer, & Schmidtke, 2004). Indeed, this will occur on 50% of task alternations with a repeated stimulus in a two-choice task. This sort of *partial repetition* entails a cost, because the repeated stimulus (7) biases a repeated response (left), which is incorrect for the current trial. The same sort of benefits and costs are observed in non-task switching environments (Frings, Rothermund, & Wentura, 2007; Giesen & Rothermund, 2014; Hommel, 1998). Because there are more easy transitions (e.g., complete repetitions) with a task repetition than with a task alternation, this produces systematic biases. Similar biases exist in congruency sequence effects (Hazeltine & Mordkoff, 2014; Hommel, Proctor, & Vu, 2004; Mayr, Awh, & Laurey, 2003; Mordkoff, 2012; Schmidt & De Houwer, 2011; Schmidt, De Schryver, & Weissman, 2014), proportion congruent effects (Risko, Blais, Stolz, & Besner, 2008), and negative priming (Rothermund, Wentura, & De Houwer, 2005).

Relatedly, response repetitions always entail a decision repetition when the task repeats (e.g., “odd” → left, followed by “odd” → left), but always entail a decision alternation when the task alternates (e.g., “odd” → left, followed by “<5” → left). Together, this implies a larger response repetition benefit on task repetitions (i.e., decision-response “complete repetition”) relative to task alternations (i.e., decision-response “partial repetition”), as has been observed in the switch cost literature (Altmann, 2011; Arrington & Logan, 2004a; Druey & Hübner, 2008; Hübner & Druey, 2006, 2008; Kleinsorge, 1999; Meiran, 2000; Rogers & Monsell, 1995; Schneider & Logan, 2005; see also, Longman, Milton, Wills, & Verbruggen, 2018, for work on decision-response learning). The same type of interaction between task switching and response repetition is also observed with the psychological refractory period (PRP) paradigm (Lien, Schweickert, & Proctor, 2003; Schuch & Koch, 2004).

Thus, the number of ways in which feature integration biases can systematically inflate the “true” switch cost (read: cost of switching the task itself) is complex. In all three of the above examples, feature integration biases systematically speed task repetitions and/or slow task alternations, thereby inflating the estimate of the cost of repeating the task itself. The contribution of feature integration biases to switch costs is particularly complex if one considers the full orthogonal combination of cue (colour) repetitions, stimulus (digit) repetitions, decision/classification repetitions, and response (key) repetitions. Schmidt and Liefooghe (2016) provided just such a breakdown of a cued task switching paradigm. Not only did they observe clear feature integration biases, but these biases systematically inflated the switch cost. However, the entirety of the switch cost was not accounted for with feature integration biases alone, a point to which we will return later. Schmidt and Liefooghe (2016) proposed that the switch cost is mainly induced by feature integration biases and much less by task-set control. The present study offers a formal implementation of this idea by modelling cued task switching via episodic storage and retrieval processes. Our exemplar-based model produces the switch cost primarily as an indirect result of feature integration biases that emerge from memory. In addition, task-set control is formalized in a very parsimonious way and, as will be discussed in further detail later on, often does not account for key effects in task switching.

### The Present Work

The rest of the paper is divided into sections. In the Model Description section, we explain the basics on how our exemplar-based memory model (PEP) works and how it has been adapted to remember and implement task instructions and to deal with multiple task situations. The Application to Task Switching section explains how the PEP model is able to explain a range of key findings in the task switching domain based on the principle of similarity-based retrieval of recently-encoded events. In particular, we will show how feature integration effects (and task-rule congruency effects) naturally emerge via memory retrieval. Finally, we present a series of simulations. In Simulation 1, we model the above-mentioned cued task switching data of Schmidt and Liefooghe (2016) and a range of other observations in the task switching domain (e.g., task-rule congruency effects). Although we certainly do not wish to claim that our model is complete enough to explain all aspects of behaviour in all variants of task switching procedures, we will attempt to show how a great deal of the complexity in task switching data can be coherently understood from an episodic framework. Moreover, we will present some novel predictions unique to our account. In Simulations 2–5, we will demonstrate how existing competing models fail to account for the binding biases that the PEP model naturally reproduces as a byproduct of episodic encoding and retrieval. The goal of these simulations is to show the notable differences in the way the PEP model produces findings from the task switching literature relative to other models. In Simulation 6, we demonstrate that the PEP model has fixed qualitative predictions (i.e., it cannot simply “fit” to any pattern of results).

## Model Description

There are numerous instantiations of what is alternatively referred to as *episodic, instance*, or *exemplar* models of memory (Hintzman, 1984, 1986, 1988; Nosofsky, 1988a, 1988b; Nosofsky & Palmeri, 1997; Nosofsky, Little, Donkin, & Fific, 2011; Medin & Schaffer, 1978; Logan, 1988). It is worth mentioning that the term “episodic” as used here and elsewhere in various literatures does not refer to episodic memory as typically conceptualized (i.e., Hippocampal-supported memory of personal experiences, etc.; Tulving, 1972), which can be dissociated from more procedural memory (Nadel & Moscovitch, 1997). The core notion of episodic models is that each experience is stored as a new memory trace. Subsequent retrieval of memory traces based on similarity (e.g., “3” retrieves memories of events where “3” was observed) allows for retrieval of the actions previously performed for the stimulus (e.g., left key). Episodic models are particularly fruitful in explaining behaviour in a range of domains (e.g., categorization, memory recall and recognition, etc.). The exact implementation varies from model to model, but the same core concepts underlie the general class of models. In this paper, we discuss the Parallel Episodic Processing (PEP) model (Schmidt, 2013a, 2013b, 2016a, 2016b, 2018; Schmidt, De Houwer, & Rothermund, 2016; Schmidt & Weissman, 2016). We expand on this particular framework because it is well suited to the simulation of response time (in addition to error rate) data.^2^

Full description of the math of the PEP model is presented in Appendix B, and full, documented source code is available for download from the website of the lead author (leadserv.u-bourgogne.fr/~jschmidt/PEP/). Here, we work through the model conceptually. Response times are simulated in the following manner. On each trial, Input nodes for presented stimuli are stimulated, and activation rises for these nodes over time on each processing cycle (a processing cycle is, roughly, a simulated millisecond of time). Activation spreads throughout the network until activation for one of the Response nodes exceeds the response threshold. How many cycles it took to reach the threshold is the simulated response time. Due to noise in the system, errors are possible if the wrong response is activated.

Most critical for the PEP model is the episodic store, which is responsible for most of the interesting phenomena that the model recovers. The model learns both when and what to respond via the storage and retrieval of memories of past events. As with all exemplar-based models, each experienced event is stored in a new memory trace. Each new memory links together the stimuli that were presented, the decision and response that were made, and (though less relevant for the present report) temporal (time-based) information. Thus, the memory store does not store *regularities* across events (e.g., “3” *tends* to be responded to with a left response), but rather a series of distinct events.^3^ Regularities across events, however, do influence behaviour via processes that operate at retrieval. For instance, each time the model responds to “3” with a left key response, a new episode is formed linking “3” to the left response. Over time, more and more such episodes will accumulate. Presentation of “3” again will lead to retrieval of these episodes, which in turn jointly activate the encoded response (i.e., left), thereby facilitating a fast left keypress response. Among other things, this allows the model to produce practice curves (see also, Logan, 1988; Newell & Rosenbloom, 1981), where initially slow performance improves across trials toward an asymptotic ideal (see also, Heathcote, Brown, & Mewhort, 2000; Myung, Kim, & Pitt, 2000).

The selfsame learning mechanism not only automatizes relevant stimulus-response links (i.e., links that are part of the to-be-performed task), but also produces a range of other diverse effects as a consequence of the same mechanism. This includes learning of irrelevant stimulus-response contingencies, stimulus-response binding effects, acquisition curves, mixing costs,^4^ and a range of findings from the attentional control domain in a single fixed-parameter model (see Schmidt et al., 2016, for simulations).

In the PEP model, older episodes (particularly those most similar to the current event) are weakened with each retrieval, such that recently-encoded episodes have more influence on behaviour than older ones. This is desirable, because newly-learned information does not get “drowned out” by a mass accumulation of old episodes. For example, while you may have memories of the many places that you have previously parked your car in your work parking lot, you want to be able to retrieve the place that you have *just* parked your car most strongly, which requires partially “forgetting” (i.e., making less retrievable) the places you have parked it before. This heavier weighting of recent events over old ones is consistent with the rapid learning observed in implicit learning paradigms (Lewicki, 1985; Lin & MacLeod, 2018; Nissen & Bullemer, 1987; Schmidt & De Houwer, 2016b) in addition to the large effects of just-encountered trials on performance in stimulus-response binding paradigms (e.g., Frings et al., 2007; Hommel, 1998; Rothermund et al., 2005).^5^

Retrieval in the model is also proportional, which means that if one, for example, response is being strongly activated via memory retrieval, retrieval of other responses will be proportionately lower. That is, the total amount of retrieval activation for all responses (or decisions, etc.) is limited, and retrieval might therefore be described as partially competitive. This is important for the stability of the model and is typical in related models (e.g., Hintzman, 1984). Although this proportional retrieval is not critical for the main aim of the present report, we will return to this point in other places throughout the manuscript where relevant for understanding the model function.

The previous version of the PEP model, however, was only able to handle single-goal tasks (i.e., where each stimulus is only associated with one decision and response). The present version of the model has been restructured to handle multi-goal situations, as in task switching. The key change to the model that allows balancing of multiple goals also allows the model to remember and implement task instructions. Similar to the way in which task switching theories propose constant reconfiguration of the cognitive system, following initial task instructions has been argued to require initial cognitive control to configure the cognitive system for the specific task requirements (Cohen-Kdoshay & Meiran, 2019; Rabbitt, 1997).

Formal (computational) and informal (verbal) models of cognition often do not clearly specify how this configuration occurs (e.g., by referring to setting up “processing pathways” or “cognitive control loops,” which is sometimes criticized as vague or “homuncular”) or do not consider task configuration at all (for a discussion, see Verbruggen, McLaren, & Chambers, 2014). Instead, focus is typically on what cognitive processes are affecting behaviour afterwards (with some exceptions; e.g., Haazebroek, Raffone, & Hommel, 2017; Haazebroek, van Dantzig, & Hommel, 2011; Norman & Shallice, 1986; Ramamoorthy & Verguts, 2012). For instance, task instructions are often “hardwired” into computational models with instruction-consistent connections between stimuli and responses, cues and goals, etc., and the question of how the system wired itself in that way is typically outside of the scope of most models.

In the new version of the PEP model, we conceptualize such configuration by appealing to simple episodic storage and retrieval processes. Instead of hardwiring instructions, as was done in the previous version of the PEP model, the new PEP version encodes memory traces of the instructed mappings (i.e., decision-to-key mappings and cue-to-task mappings), as a participant will do via, for instance, memory rehearsal or mental imagery (Ruge & Wolfensteller, 2010; see also, Jeannerod, 2001; Jeannerod & Frak, 1999; O’Shea & Moran, 2017; Theeuwes, Liefooghe, De Schryver, & De Houwer, 2018). This allows for automatic retrieval during subsequent execution of the task (Brass, Liefooghe, Braem, & De Houwer, 2017; Martiny-Huenger, Martiny, Parks-Stamm, Pfeiffer, & Gollwitzer, 2017). In this way, remembering and implementing instructed stimulus-response mappings and goals can be achieved via the same episodic encoding and retrieval mechanisms used to support event binding and learning through experience, which is consistent with neuroscience work on mental preparation (e.g., Demanet et al., 2016; for a review, see Brass et al., 2017). Indeed, instruction following may simply be regarded as a form of covert learning in our model, consistent with the observed beneficial effects of mental practice on subsequent performance (for a review and meta-analysis, see Driskell, Copper, & Moran, 1994).

Although memory retrievability for older memories decreases with each retrieval in the PEP model (including for instruction-based memories), instructions will not be “forgotten.” In particular, each time the system retrieves the appropriate response to a stimulus and executes it (e.g., odd → left), the instructed relation is effectively re-encoded in the newly-formed episode. Thus, the system is self-perpetuating. Some added provisions were made to keep the model responding correctly after errors (see Appendix B), but these provisions are not crucial for the main task switching phenomena of interest.

Unlike previous versions of the PEP model, the newly-added feature allowing the model to follow instructions also allows the model to implement multiple task goals, allowing simulations of task switching. In particular, the model is able to retrieve from memory what task to perform to which cues (in addition to which keys to press for which decisions). More importantly, the fact that memory retrieval is particularly biased by recently-encoded events produces feature integration effects. For instance, if a blue cue was just encoded, then re-presentation of the same cue on the next trial allows for faster retrieval of the appropriate goal. The same applies to repetitions of stimuli or decisions. These feature integration biases are the primary means via which the PEP model explains task switching behaviour, rather than task-set control.

## Extension to Task Switching

There are two important features of the PEP model that are interesting in the context of task switching. First, the extended version of the PEP model that we introduce in this paper is able to remember and implement instructed arbitrary stimulus-response mappings and cued task goals. This expansion of the PEP framework makes it possible for us to simulate more complex situations that include multiple task goals, like task switching. The PEP model “configures” itself via episodic retrieval, by: (a) *retrieving* from memory the task goal on the basis of a cue, and (b) retrieving an arbitrarily instructed response for the stimulus on the basis of the current goal. Strictly speaking, the model does not do anything different on a task repetition versus alternation. On each trial, the model simply sees the cue and retrieves from memory the associated goal. As we will elaborate on in the General Discussion, this memory operation could be considered as a form of task-set control. However, such control occurs in the same way whether or not a task changes. Thus, using terminology such as “task-set repetition” versus ‘task-set reconfiguration” on, respectively, task repetitions and task switches would be inaccurate within PEP. As will be elaborated later, this two-step retrieval allows PEP to be able to achieve both tasks. However, it is far less important than many competing accounts might suggest for producing the key effects in task switching paradigms, including the switch cost itself.

More concretely, like previous versions of the PEP model, the new model has Input nodes for stimuli (cues and targets), Decision nodes for the classifications, and Response nodes for the keys. Crucially, Goal nodes were also added. They activate like regular nodes and boost the sensitivity of the relevant Decision nodes. For instance, the parity Goal node sensitizes the odd and even Decision nodes. The sensitivity of a Decision node determines the rate at which incoming input from other nodes (e.g., stimulus Input nodes) increases the Decision node activation level. In this way, presentation of a cue leads to retrieval of the goal, which prepares the system to choose between the sub-options of the goal (e.g., odd vs. even with a parity goal). The Decision nodes for a non-active goal (e.g., “<5” and “>5”) can still receive input, but activate much less rapidly (i.e., lower sensitivity to input). Together, these changes allow the model to simulate performance in environments in which the task requirements change from trial to trial (e.g., task switching).

In experiments with two or more cued tasks, participants need to decide both which task to implement based on the cue and which response to make to the stimulus based on this goal (Mayr & Kliegl, 2000, 2003; Rubinstein, Meyer, & Evans, 2001; cf., Logan & Bundesen, 2003, 2004; Schneider & Logan, 2005, who argued against this two-step structure of processing). This is reflected in the model by changes in search strengths over the course of the trial. In particular, memory search strength from the cue to the goal is temporarily stronger at the start of the trial. After one of the goals is sufficiently active, the cue-based goal search is weakened and search strength for the decision and response on the basis of the target is strengthened. After trial completion, all searches are weakened again. In other words, while retrieval to and from all node types is continuous, the two-stage nature of task-set control is captured by increased search strength of the currently sought-after information. For tasks in which there is only one fixed goal this two-stage search is less relevant. This two-stage search is similar to the serial prioritization of stimuli in the ECTVA theory of visual attention (Logan & Gordon, 2001) as applied to dual tasking. Together with the above-mentioned adaptations, these changes allow the model to successfully implement multiple goals in task switching. However, we again highlight that the two-stage search is not responsible for the actual effects (i.e., differences in condition means) that we will investigate.

The second and more crucial aspect of the PEP framework for present purposes is that recently-encoded episodes are more strongly retrievable from memory than older memories, as described in the Model Description section. A logical consequence of this “learning with decay” mechanism in the PEP model is the emergence of feature integration effects (Schmidt et al., 2016). Information that was *just* encoded on the immediately-preceding trial has a large impact on what is retrieved from memory, because the just-encoded memory has experienced little or no decay.^6^ The reason why this works is that if a stimulus (e.g., 7) was just presented with a given response (e.g., left key), then repetition of the same stimulus on the immediately following trial will activate the just-encoded episode, which in turn activates a repeated response. If the (new) correct response is the same (left), then responding will be facilitated. However, if the response needs to change (right), then the retrieval bias in favor of the repeated (but now incorrect) response (i.e., left) slows selection of the correct response (right). This is due to the competitive retrieval explained in the Model Description section. The same logic follows for repetitions versus alternations of cues, target stimuli, decisions, or responses: complete repetitions facilitate performance, and partial repetitions entail a cost. In the simulations, we show how these feature integration biases influence performance in a cued task switching experiment, and how they confound^7^ the measure of the switch cost and various other key findings in the task switching domain.

## Simulation 1: Feature Integration

Schmidt and Liefooghe (2016) used the same setup described in the examples above, in which participants responded to digits (1–9, excluding 5) with either parity (odd vs. even) or magnitude (<5 vs. >5) decisions. The task was determined by a colour cue (blue, red, green, or yellow), two of which were assigned to one task and two to the other task. Cues were presented 200 ms in advance of the digit, and the response-cue interval was 500 ms. This cue interval was used because switch costs are largest with short cue-target intervals. Responses were made with left or right key presses. Assignment of odd and even was counterbalanced across participants to keys, whereas <5 and >5 were always assigned to the left and right keys, respectively. Participants had 3000 ms to respond, with feedback after errors. The two tasks were equiprobable on all trials to avoid learning biases (see Logan et al., 2007).

In their study, trials were split up on the basis of each type of repetition or alternation of stimuli, decisions, responses, cues, and tasks (see Table 1 for a list of the conditions). There were task repetitions with cue repetitions (cue) and task repetitions without cue repetitions (rep). Task alternations (alt) always involved alternating cues. The next two letters in the condition names refer to whether the stimulus (first letter) and the response (second letter) repeats (R) or alternates (A) from the previous trial. For instance, “cue-AR” refers to a trial in which the cue/task repeats, the stimulus alternates, and the response repeats. Note that response key repetitions also involve a decision (classification) repetition during a task repetition (e.g., “odd” → “odd”), whereas a response (key) repetition involves a decision *alternation* on a task switch (e.g., “odd” → “<5”). Thus, a major result of this is that it is difficult to compare task repetitions and alternations with a response repetition. In fact, the only two conditions that differ *only* in the repetition versus alternation of the task are rep-AA and alt-AA, highlighted in grey in Table 1, which is a purer measure of the cost of switching the task only (with caveats we will highlight in the General Discussion).

**Table 1:** Trial types from Schmidt and Liefooghe (2016) with example trials.

Condition	Repetition Type					Trial	

	Task	Cue	Stimulus	Decision	Key	Preceding	Current

cue-RR	✓	✓	✓	✓	✓	blue-3-odd-left	blue-3-odd-left
cue-AR	✓	✓	✕	✓	✓	blue-3-odd-left	blue-1-odd-left
cue-AA	✓	✓	✕	✕	✕	blue-3-odd-left	blue-2-even-right
rep-RR	✓	✕	✓	✓	✓	blue-3-odd-left	red-3-odd-left
rep-AR	✓	✕	✕	✓	✓	blue-3-odd-left	red-1-odd-left
rep-AA	✓	✕	✕	✕	✕	blue-3-odd-left	red-2-even-right
alt-RR	✕	✕	✓	✕	✓	blue-3-odd-left	green-3-<5-left
alt-RA	✕	✕	✓	✕	✕	blue-7-odd-left	green-7->5-right
alt-AR	✕	✕	✕	✕	✓	blue-3-odd-left	green-2-<5-left
alt-AA	✕	✕	✕	✕	✕	blue-3-odd-left	green-6->5-right

**Figure 2:**
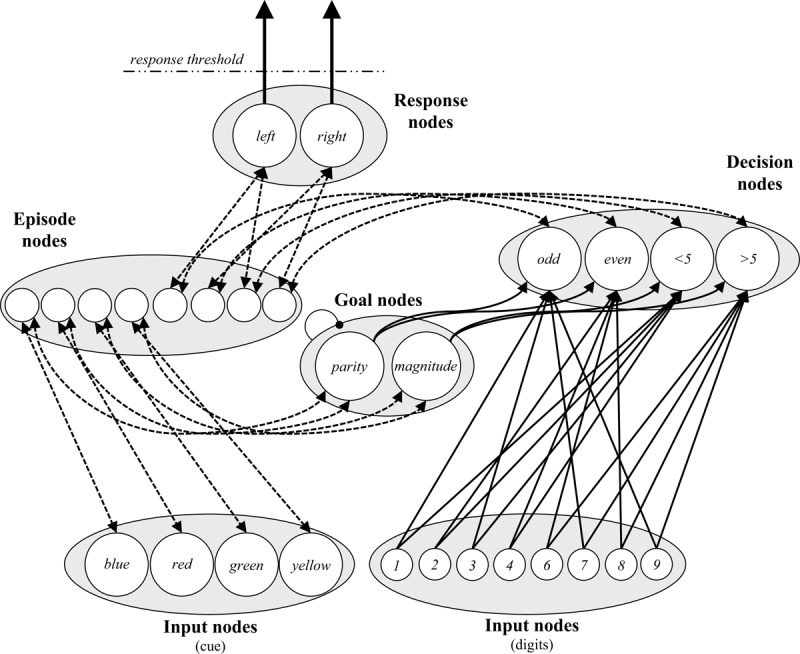
The Parallel Episodic Processing (PEP) model as it applies to cued task switching. Instructed cue-goal and decision-response mappings are illustrated with dashed lines. Hardwired connections are illustrated with solid lines. Note that *all* node types are connected to episodes during encoding of experienced events, but this is not illustrated for simplicity. Connections ending in arrows are facilitative and connections ending in circles indicate within-layer competition (Goal nodes only).

Figure 2 displays the model as it applies to the cued task switching experiment of Schmidt and Liefooghe (2016). Episode nodes do recursively connect to *all* types of nodes during the task, but the figure indicates the overlearned connections as solid lines. The model also stores the task instructions (i.e., stimulus-response and cue-goal mappings) at the start of the simulation, indicated with dashed lines. Thus, the model “knows” which numbers are odd or even, which numbers are greater or less than five, that “odd” and “even” are parity decisions, and that “<5” and “>5” are magnitude decisions (i.e., pre-existing knowledge). However, it must remember instructions about which keys correspond to which decisions and which goals have to be executed on presentation of which cues. In other words, it is assumed that participants already have knowledge of concepts like “odd,” “even,” “parity,” etc. in their long-term memory and that these conceptual elements can be bound together while processing task instructions to allow for subsequent retrieval (for a related notion, see Meiran, Cole, & Braver, 2012). This allows the model to maintain the task rules throughout task execution.

At the start of the simulation, the model stores one memory each for each of the instructed cue-goal links (i.e., blue-parity, red-parity, green-magnitude, and yellow-magnitude) and one memory each of the instructed decision-response links (i.e., odd-left, even-right, <5-left, >5-right). During task execution, the goal is retrieved on the basis of the presented cue, initially by retrieving the initial instruction memory, later via a combination of the initial instruction memory (which decays) and memories of experienced cue-goal co-occurrences. Similarly, after making a decision (e.g., “odd”), the key to press is retrieved from memory search. Again, this is initially based on the instruction memory, then later primarily on the basis of experienced decision-response co-occurrences.

A “sample” of 500 simulated participants was run. This is more than enough “participants” to ensure accurate description of model predictions (i.e., very, very small standard errors), so no statistics are reported. However, all discussed differences are statistically significant, typically by a sizable margin. In our past projects with the PEP model, we stuck with a fixed model parameterization and focused only on rough qualitative fit. However, we also made attempts to improve quantitative fit to data in the present report. This is much less straightforward than in the simpler task switching models that we will discuss later, as many parameter-fitting techniques are unfeasible in models with a large parameter space and high computational “cost” (i.e., simulation time) of testing a single parameterization. Evolutionary algorithms are well suited to fitting complex models, however, so we programmed one (see Appendix C) to fit several of the model parameters to the participant data. It is important to stress, however, that the *qualitative* (i.e., directional) predictions of the model are quite invariant across parameterizations, which we will demonstrate in Simulation 6.

The stimuli and procedure “presented” to the model were identical to that for the participant sample in Schmidt and Liefooghe (2016). In particular, each “participant” was presented 800 trials, selected randomly with replacement. On each simulated trial, the model was presented a colour (cue) for 200 cycles, followed by the target digit for 3000 cycles or until a response was made. In the following, we highlight the key confounding influences that feature integration biases produce on the switch cost. In sections to follow this simulation, we show how several important findings from the task switching domain are logically entailed from the feature integration biases to be demonstrated below, and we will show this directly with the simulated data.

**Figure 3:**
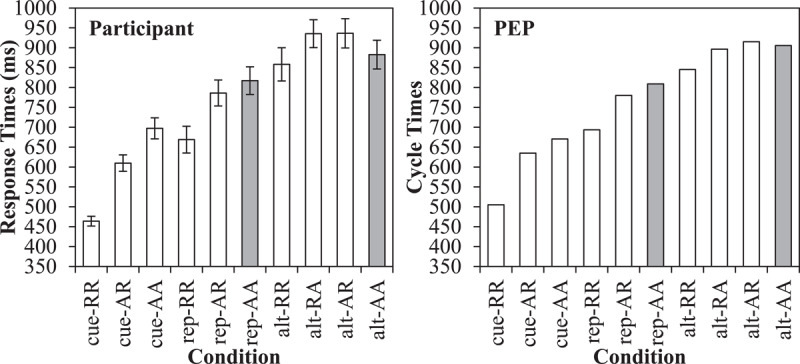
Cued task switching effects, including original response times (with standard errors) from Schmidt and Liefooghe (2016) and Simulation 1 cycle times from the PEP. The grey bars indicate the true switch cost.

The mean cycle times are presented in Figure 3 in the right panel, along with the original participant data in the left panel. Firstly, cue repetitions (603 cycles) were faster than task repetitions (761 cycles), as in the participant data. For cue repetitions, there was both a stimulus repetition benefit for cue-RR (505 cycles) relative to cue-AR (635 cycles) of 130 cycles and a response repetition benefit for cue-AR relative to cue-AA (671 cycles) of 36 cycles. For task repetitions (with cue alternations), the stimulus repetition benefit for rep-RR (693 cycles) relative to rep-AR (780 cycles) was also present but (as expected) smaller at 87 cycles. The response repetition benefit of 29 cycles for rep-AR relative to rep-AA (809) was robust in the model, but small. This is similar to the participant data, where the response repetition benefit was small (same direction) and not significant. Note critically that relative to a complete alternation (rep-AA) repeating the response, the stimulus and response, or the cue all produce facilitative effects.

For task alternations (switches), partial repetitions (906 cycles) were slower than complete repetitions/alternations (875 cycles). In particular, complete repetitions (alt-RR; 845 cycles) were faster than partial repetitions where only the stimulus (and not the response) repeats (alt-RA; 896 cycles). Similarly, complete alternations (alt-AA) were robustly faster (905 cycles) relative to partial repetitions where only the response (and not stimulus) repeats (alt-AR; 936 cycles). Thus, the standard binding interaction is observed, as in the participant data. The cost for alt-RA trials is not large (but see the errors). Also of importance, there was still a difference between alt-AA and rep-AA trials, indicated in grey in the figure. This is interesting because it is a “pure” switch cost independent of cue, stimulus, decision, or response repetitions. Note that relative to these reference conditions, feature integration biases consistently *speed* performance on task repetitions, but not on task alternations, systematically inflating the switch cost.

**Figure 4:**
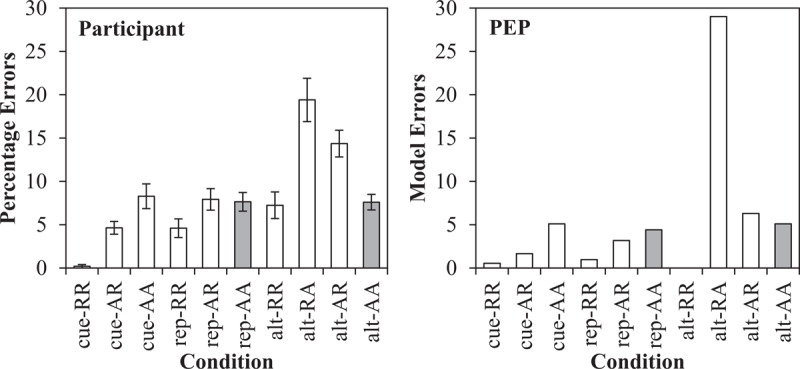
Cued task switching effects, including original error rates (with standard errors) from Schmidt and Liefooghe (2016) and Simulation 1 errors with the PEP. The grey bars indicate the true switch cost.

The simulated error data are presented in Figure 4 on the right panel, along with the participant data on the left. The model only failed to respond on 1.7% of the trials, and these trials are excluded. The condition means (left to right on Figure 4) were 0.5, 1.7, 5.1, 1.0, 3.2, 4.4, 0.0, 29.0, 6.3, and 5.1%. For brevity, we note only that the errors produced the same results (robustly) as the cycle times, though error rates were generally much lower than in the participant sample (as is often the case in other models), with the exception of the alt-RA condition. The “pure” switch cost measure (alt-AA – rep-AA) was trivially in the wrong direction in the simulated errors. In the participant data, the numerical difference was almost exactly zero (also only a fraction of a percentage in the simulated data). Thus, the simulated error data match reasonably well with the original participant data, however imperfectly.

## Extra Analyses: Switch Costs, Cue Repetitions, Response Repetitions, and Congruency

Simulation 1 presented a relatively complex dataset in which a cued task switching procedure was decomposed into ten unique sub-conditions in order to demonstrate that feature integration effects emerge from simple episodic encoding and retrieval processes. As we will expand on in the following, the simulation results also showed how feature integration biases systematically *speed* task repetitions, but systematically *slow* task alternations. Within this complex dataset a range of other key task switching phenomena are present, though perhaps not in an easily-visible and self-evident way. In that vein, in this section we will show that these other important task switching effects are present in the already simulated data. For simplicity, we focus only on simulated response times, but error rate data always produced the same pattern of results. In each subsection, we will explain how the given effect emerges from episodic feature integration biases.

### Switch Cost

**Figure 5:**
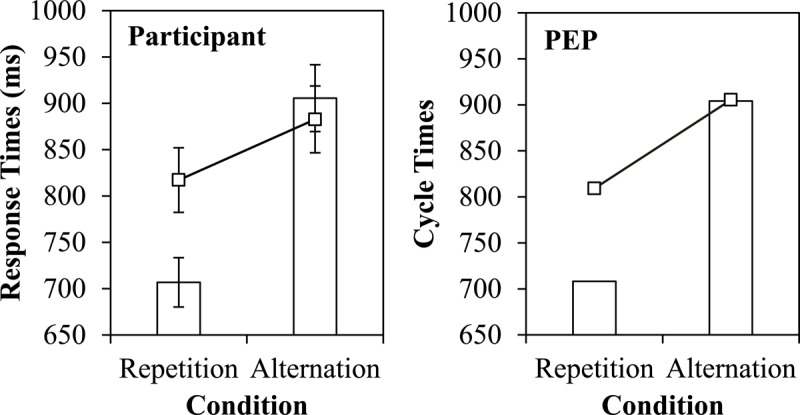
Simple switch cost contrast (bars) and unconfounded switch cost (line) in participant response times and PEP-simulated results.

In Figure 5, the simple switch cost for both the simulated and participant data are presented (bars). Presented this way, it may appear as if the model is substantially impaired by reconfiguration of the task set (or goal) on task switches, with a 196 cycle switch cost. However, it is critical to note that this effect, at least primarily, does *not* emerge because of a cost of switching the task itself. This can be observed by the line on Figure 5, indicating the switch cost remaining after excluding the contribution of feature-integration effects, which is notably smaller. Task repetitions are fast because they can: (a) contain cue repetitions and (b) include both response repetitions and complete (stimulus + response) repetitions in which the repeated key press (e.g., left key) also corresponds to a repeated decision (e.g., “<5”). Both (a) and (b) speed responding, and neither can occur for a task switch. Task switches are additionally slowed by the trials in which a stimulus is repeated, but a change in the response key is required, which is not possible on a task repetition. There is a remaining effect (at least in response times) when these biases are excluded (see General Discussion for implications), but much of the switch cost here results from repetitions of cue, target, classification, and response bindings in episodic memory.

To clarify why a switch cost remains after controlling for binding biases in the model, we conducted a second simulation with another 500 simulated participants. This second simulation was identical to that already reported with one exception: during the 500 cycle response-cue interval (i.e., the time between response execution and cue onset) the activations of the two goal nodes were set to the mean of the two activations. In other words, goal “inertia” from the previous trial was effectively shut off. This reduced the “true” switch cost measure substantially to 8.5 cycles. Though still “significant,” this was a 91% reduction. Thus, carryover activation of the previous task goal explains the “true” switch cost in the PEP, at least in the current instantiation (but see General Discussion for alternative possibilities).

### Cue Repetition Benefit

**Figure 6:**
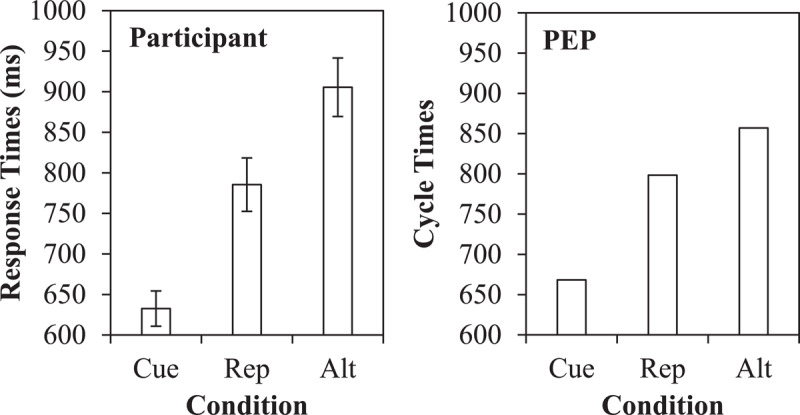
Cue repetition benefit data, with participant response times and simulated results from the PEP.

As already discussed, cue encoding can benefit from repeated cues, which can only occur on a task repetition. Although potentially one of the easiest-to-see components in the Simulation 1 data, Figure 6 presents the data broken down as a function of task repetitions with cue repetitions (cue), task repetitions without cue repetitions (rep), and task switches (alt). As can be observed, the majority of both the simulated and participant switch costs are attributable to faster responses when the cue repeats (668 cycles) relative to when just the task repeats (798 cycles). That is, the model is faster when the cue colour repeats, and this is because the model can more quickly retrieve the goal linked to this cue due to the just-encoded episode linking the cue to the goal. When the cue does not repeat, there is still a difference between task repetitions and alternations (857 cycles). However, this remaining 59 cycle switch cost is still exaggerated due to the contribution of other feature integration biases (i.e., stimulus, decision, and response binding effects, as discussed earlier).

### Response Repetition Benefit and Asymmetry

As previously discussed, repeating a response generally benefits performance, but this is more the case for task repetitions (where a robust response repetition effect is observed) than for task switches (where small or even reversed response repetition effects are often observed). This interaction has been interpreted in a number of ways (for a discussion, see Druey, 2014). According to one account (Kleinsorge, 1999; Kleinsorge & Heuer, 1999), reconfiguring the task-set on a task alternation leads to a bias to also alternate the response as well, which entails a cost when a repeated response *is* needed (e.g., negating any simple repetition priming). According to another account, response repetition costs for task switches are due to active inhibition of the just-made response (Druey, 2014; Hübner & Druey, 2006). In the PEP model, however, response repetition effects are produced by memory retrieval (see also, Altmann, 2011; for a similar idea, but phrased in terms of associations rather than memory traces, see Rogers & Monsell, 1995). This is related to a recent study by Koch, Frings, and Schuch (2018), where it was demonstrated that response repetition benefits were modulated by overlap in cue modality, which the authors similarly argued as evidence for binding contributions to response repetition effects.

**Figure 7:**
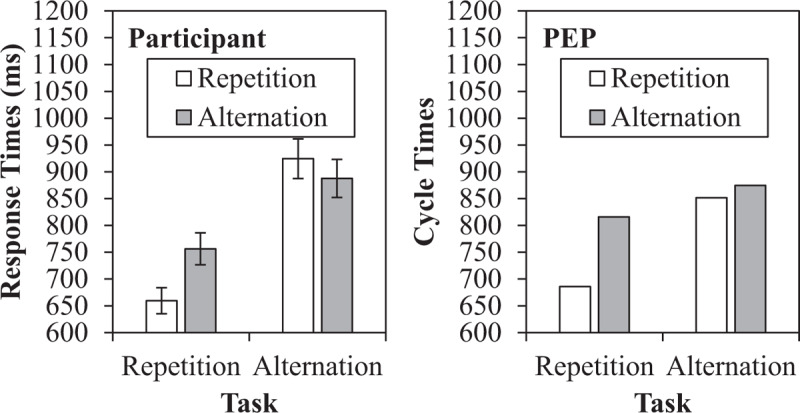
Response repetition benefit data as a function of task repetition, with participant response times and simulated results from the PEP.

Perhaps more difficult to see in the Simulation 1 data than the cue repetition effect is the response repetition effect, and how it differs across task repetitions and alternations. These data are presented in Figure 7. As can be observed in the simulated data, repeating the response produces a benefit to responding, but this is much more pronounced for task repetitions (130 cycles) than task switches (23 cycles). This is, in part, due to the fact that repeating a response on a task repetition entails making the same decision/classification (e.g., “odd”) twice in a row (a clear advantage), whereas two different classifications are made on a response repetition during a task switch (i.e., a response repetition advantage minus a classification-response inconsistency). For instance, after encoding an “odd” decision bound to a left response, a second “odd” decision will allow for rapid retrieval of a left response via this just-encoded episode. However, if the second decision is “<5,” then choosing a left response will *not* benefit from the just-encoded odd-left episode.

Because we did not report on response repetition effects in our original paper (Schmidt & Liefooghe, 2016), we note that the response repetition benefit (97 ms) is significant for task repetitions in the participant data, *t*(24) = 9.157, *SE_diff_* = 11, *p* < .001, *η^2^* = .78, and significantly reversed for task alternations (37 ms), *t*(24) = 4.087, *SE_diff_* = 9, *p* < .001, *η^2^* = .41. The interaction is also significant, *F*(1,24) = 87.685, *MSE* = 2562, p < .001,\,\eta _p^2 = .79. Thus, the model appropriately produces switch-modulated response repetition effects, but the direction of the response repetition effect for task switches is incorrect. A response repetition cost is not always observed in participant samples, however (see Altmann, 2011, for a discussion), and, perhaps related, the direction of this effect is one of the few things that is parameter dependent in the PEP model. Whereas the model will always produce an asymmetry between task and response repetitions (i.e., due to unambiguous benefits for task repetitions), inconsistency in whether the response repetition effect in task alternations is positive, negative, or null is not so surprising from the perspective of the current framework. This is because the response repetition contrast involves a complex mix of four trial types. Response repetition trials include alt-RR trials (where the stimulus-response binding is consistent with the previous trial, but the stimulus-decision and decision-response bindings are inconsistent) and alt-AR trials (where the stimulus-response and decision-response bindings are inconsistent). Response alternation trials include alt-RA trials (where the stimulus-decision and stimulus-response bindings are inconsistent) and alt-AA trials (where the stimulus, decision, and response features are non-repeated). Thus, which of these biases in this complex web outweigh the others (which could be highly variable from one design to another) will determine the direction of the response repetition effect for task alternations. However, the direction of the response repetition effect for task switches becomes *unambiguous* when stimulus repetitions are prevented in the procedure and/or removed from analyses, as is frequently the case in assessments of response repetition effects (e.g., Druey & Hübner, 2008; Hübner & Druey, 2006, 2008). This happens because this trim excludes alt-RR and alt-RA trials, leaving only (slow) partial repetitions with a response repetition (alt-AR) and (fast) complete alternations with a response alternation (alt-AA). As can be observed in Figures 3 and 4, the PEP model *does* produce a response repetition cost when analysed this way. Note also that the response repetition cost is partially absent in simulated response times, at least in part, because the alt-AR cost was more pronounced in errors (where there is a response repetition cost) than in response times, as mentioned earlier. Thus, the PEP model not only does a reasonable job of matching past results, but also suggests a reason why the asymmetry is not always consistent.

### Task-Rule Congruency

We now examine what happens in the model when two instructed stimulus-response mappings for one stimulus compete with each other. That is, we investigate crosstalk between two sets of instructions. Within the task switching literature, it has been demonstrated that such a situation gives rise to *task-rule congruency* effects (e.g., Meiran, 1996, 2000; Rogers & Monsell, 1995). In particular, in studies on the task-rule congruency effect, *congruent* stimuli require the same keypress response regardless of which task is executed. For instance, if both “odd” and “<5” are mapped to the left key, then “3” requires a left keypress in both cases. In contrast, *incongruent* stimuli require different keypress responses in the two tasks. Using the previous example, “7” requires a left keypress to indicate “odd,” but a right keypress to indicate “>5.” The standard finding is that responding on incongruent trials is slower and less accurate than on congruent trials. It has further been observed (e.g., Meiran, 1996; Kiesel, Wendt, & Peters, 2007), albeit not always robustly, that switch costs are larger on incongruent trials than on congruent trials (for a review, see Kiesel et al., 2010).

There are various different accounts of the origin of task-rule congruency effects. According to one view, the task rules for both tasks are held in working memory throughout the task (Meiran, 2000). The task rules for the currently-irrelevant task-set are weakened, but remain in working memory and compete with the currently-relevant task codes. This is related to the notion that conflict emerges via a *mediated route*, where the stimulus is classified on both dimensions (e.g., “7 is odd and greater than five”), leading to two competing response options for incongruent trials but not congruent trials (Kiesel et al., 2007; Schneider & Logan, 2014). Other accounts propose that only one “task-set” occupies working memory at any one time, but retrieval of long-term stimulus-response associations (including task-irrelevant associations) can still influence processing (Hommel & Eglau, 2002; Kiesel et al., 2007; Mayr & Kliegl, 2000). This is related to the notion of a *non-mediated route*, where direct retrieval of previous stimulus-response pairings from memory produces conflict (Meiran & Kessler, 2008; Wendt & Kiesel, 2008). Task-rule congruency effects may have more than one origin (Schneider, 2015), but the distinction between these accounts is heavily blurred in the PEP model. For instance, if we consider Goal and Decision node activations as “working memory” in the PEP, how stimuli are classified in the “mediated route” is determined via retrieval from memory (“non-mediated” route), which is itself influenced both by prior pairings in the experiment (recent learning) and long-term associations (e.g., “7 is odd”).

In any case, task-rule congruency effects are a memory retrieval effect in the PEP. When the model searches for the appropriate response to a stimulus, memories of the stimulus in both the currently-relevant and currently-irrelevant task will be retrieved. For instance, the model will have many trial memories of “7.” For some of these, the decision “odd” was made (and thus encoded) along with the left response (also encoded). For other memories, “>5” and the right key are recorded. As such, when the model is presented with “7” it will retrieve both sets of episodes. If the current goal (e.g., parity) has been determined, then the participation of the goal in memory search will ensure *stronger* retrieval of the memories of “7” linked to the appropriate goal (and thus appropriate decision and response). However, the goal-inappropriate (e.g., magnitude) memories of “7” will also be retrieved (i.e., because the stimulus “7” matches these episodes). Thus, memory retrieval will be partially biased toward the wrong response on incongruent trials (i.e., some retrieval activation directed towards the goal-inappropriate response), slowing decision time and inflating errors. On congruent trials, in contrast, *all* memories of the stimulus are linked to the same response key (e.g., left), albeit also to a different decision (e.g., “odd” and “<5,” both mapped to a left response). A response can therefore be determined more readily. As such, the task-rule congruency effect results from the exact same memory processes as those that produce the feature integration biases already discussed (for a similar point, see Schmidt et al., 2016).

**Figure 8:**
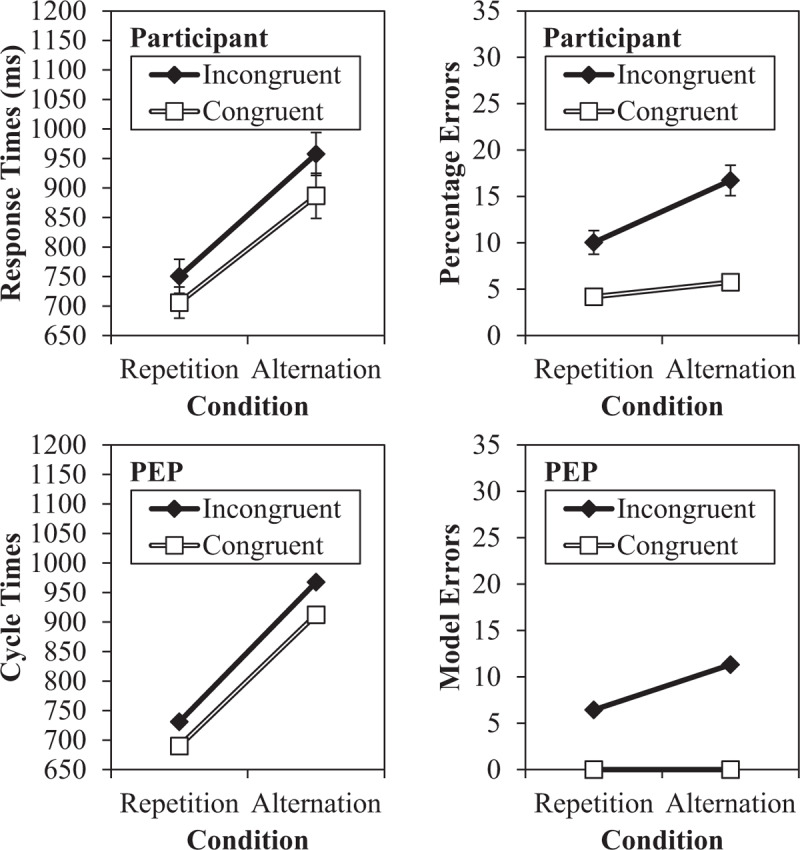
Task-rule congruency effect, including original response time and error data and simulated data from the PEP.

We can again use the already-simulated data to investigate task-rule congruency effects by recoding on the basis of congruency. The simulation results, along with the original data, are presented in Figure 8. We present error data here, as well, because task-rule congruency is not inferable from Figure 4 (unlike the preceding sections). As can be observed, the model produced a task-rule congruency effect, with incongruent trials being slower (849 cycles) and more error prone (8.9%) than congruent trials (801 cycles and 0.0%, respectively). Errors were slightly lower in the model, especially for congruent trials where errors were rare,^8^ but the qualitative fit was good. Also noteworthy, the model correctly produces a larger switch cost for incongruent trials relative to congruent trials, as observed in the participant data. The reason for this asymmetry is again feature integration biases. For instance, if an *incongruent* stimulus repeats on a task repetition, then the stimulus-response (and stimulus-decision and decision-response) binding remains consistent (e.g., “7” followed by “7,” both in the parity task and therefore with the same decision and response). However, on a task alternation the stimulus-response binding (and also the stimulus-decision and decision-response binding) *changes* for a repeated incongruent stimulus (e.g., repeating “7” but changing the task means a *different* decision and *different* response to “7” are required). In other words, feature integration biases not only confound the main effect of task repetition (switch cost), but also the *interaction* with congruency.

### Summary

The Simulation 1 data revealed several interesting observations. We were able to simulate the full pattern of means from a feature integration decomposition of cued task switching (Schmidt & Liefooghe, 2016). As the results in the Extra Analyses section revealed, the model produces a large switch cost, at least some of which we demonstrated to be due to “goal inertia,” but the majority of this switch cost is due to simple feature integration biases. As in participant data, cue repetition benefits explain a very large chunk of task repetition speeding. However, stimulus-decision, stimulus-response, and decision-response bindings explain further variance in the switch cost (i.e., further speeding of task repetitions and further slowing of task alternations). Response repetition benefits naturally emerge from repeating a response, and the asymmetry in this response repetition benefit across task repetitions and alternations is further explained by feature integration biases, most notably due to the consistent decision-response bindings on task repetitions, but inconsistent decision-response bindings on task alternations. The same memory encoding and retrieval processes that produce these more transient feature integration biases also produce more longer-term learning effects (see also, Schmidt et al., 2016). This produces task-rule congruency effects. That is, congruent stimuli are *consistently* bound to the same response in both tasks (meaning that all memories of a stimulus point to the same response), whereas incongruent stimuli are bound to different responses in each task (meaning that roughly half of the memories for a given incongruent stimulus point to one response, whereas the other half point to a different response). In this way, task-rule congruency effects are the exact same as feature integration biases, only over a longer time scale (i.e., the influence of many episodes, rather than just the immediately preceding one).

## Simulations 2–5: Comparison to Other Models

To illustrate what makes the switch cost as simulated by the PEP model drastically different than that of other task switching models, we reprogrammed a Java version of the Gilbert and Shallice (2002) model (using the backbone of the PEP model), and Excel versions of CARIS (Meiran et al., 2008), the Altmann (2011) feature integration, and the Schneider and Logan (2005, 2009, 2014) priming models. The goal of these simulations is not to argue that the PEP model has won a “competition” with other models (see Roberts & Pashler, 2000), but rather to illustrate the differences between how these models and the PEP produce task switching phenomena. Using these models, we will simulate the experiment of Schmidt and Liefooghe (as in Simulation 1). We will show that many of these models produce the switch cost just fine, but in a drastically different way than the PEP model. In order to make such comparisons easier, rescaling of simulated response times was conducted. That is, simulated response times were “stretched” to the same standard deviation between condition means as in the participant data and centered on an intercept. The CARIS and Schneider and Logan models already have parameters for this built in.

**Figure 9:**
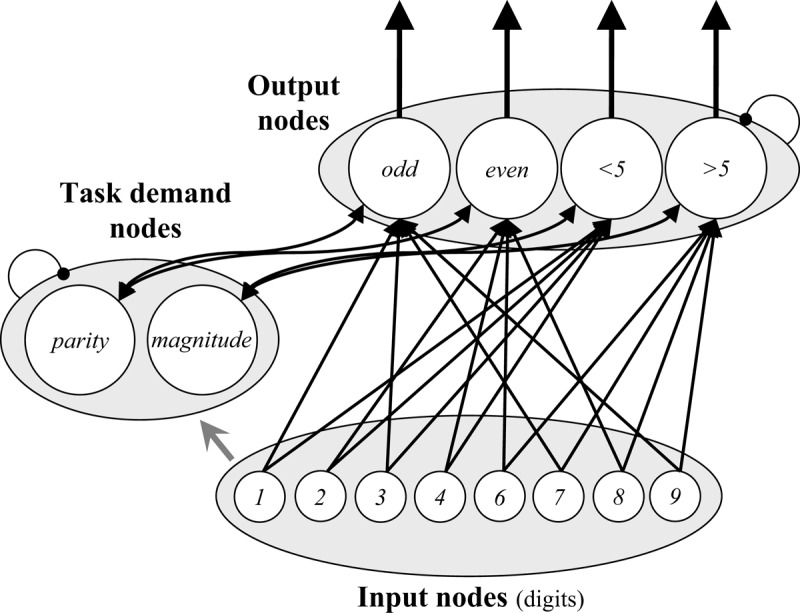
The Gilbert and Shallice (2002) model as it applies to cued task switching. All connections are hardwired. *Notes*: arrow = facilitative connection, circle = within-layer competition, grey arrow = all-to-all connections from stimuli to task demand nodes.

First, consider one of the more prototypical task switching models from Gilbert and Shallice (2002). Like most task switching models (verbal or computational), this model attributes *most* of what is going on in a task switching experiment to a legitimate switch cost. In the original report, there were two versions of the model, one with and one without stimulus-to-task connections. Though we programmed both, we only consider the better fitting full version (data from other version available on request). This model is illustrated in Figure 9. It has input nodes for the stimuli, output (decision/response) nodes for each decision, and two task nodes. The task nodes facilitate the output nodes for the relevant task. Primarily, this model assumes that carryover activation of the previous task-set leads to inter-goal conflict on task switches and task-set priming on task repetitions (i.e., task-set inertia). In particular, activations of task nodes change gradually over the course of the trial, with the currently relevant task node activation increasing and the currently irrelevant task node activation decreasing. Due to carryover activation from the prior trial, however, the relevant task node will begin the trial more active than the irrelevant one on task repetitions, and the reverse on task alternations. This task node activation advantage for the repeated task over the non-repeated task is the sole mechanism that produces the switch cost in this model. In contrast, the PEP model may have some “task set inertia” in the Goal nodes, but most of what the model produces is not due to this, but instead to the feature binding biases discussed earlier. Thus, both models produce a switch cost, but for very different reasons.

**Figure 10:**
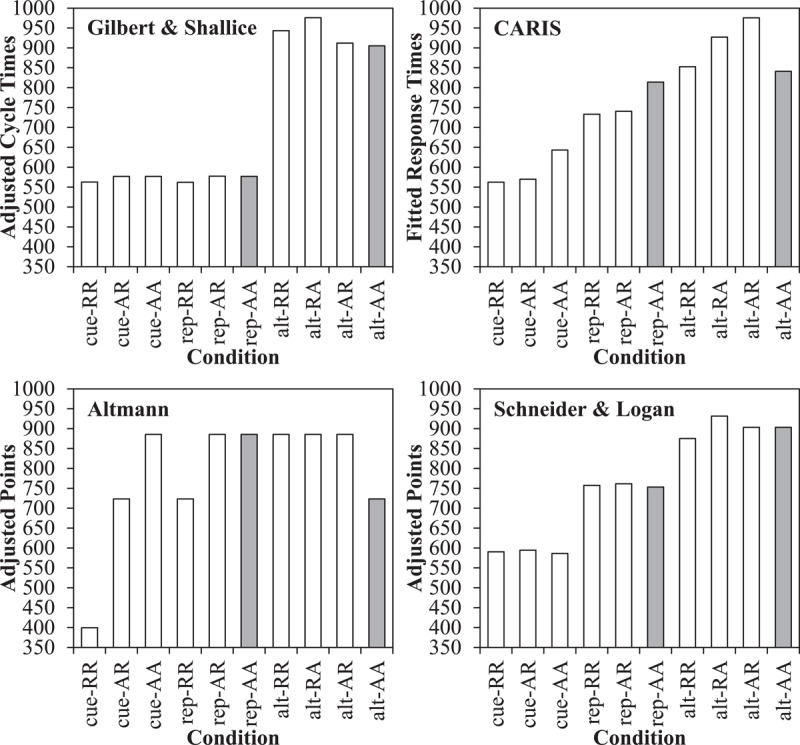
Simulations of cued task switching effects from the Gilbert and Shallice, CARIS, Altmann, and Schneider and Logan models. The grey bars indicate the true switch cost.

The model was run with 200 simulated participants. Given that the model produces almost zero variability in response times, this is more than enough to ensure accurate model fit. The results of the model are presented in Figure 10 (for comparison, see the participant data in Figure 3). Most notably, the model attributes the switch cost almost in its entirety to a true cost of switching. That is, almost all variability in simulated response times is determined by whether or not the task repeated. A very small benefit is observed for complete repetitions when the task repeats (i.e., cue-RR, task-RR), but there is no response repetition benefit for cue-AR or task-AR. There is also no difference at all for task repetitions with versus without a cue repetition. The normal binding interaction on task switches is technically present, but the qualitative ordering of condition means is clearly incorrect. Though not presented for brevity, this version of the model does produce a task-rule congruency effect, albeit a trivially small one. The error rates are not presented, because the model produces almost zero errors. These simulations demonstrate clearly the drastic difference in the way the PEP model and traditional task switch theories explain the switch cost (i.e., as a binding effect and as task-set inertia, respectively).

**Figure 11:**
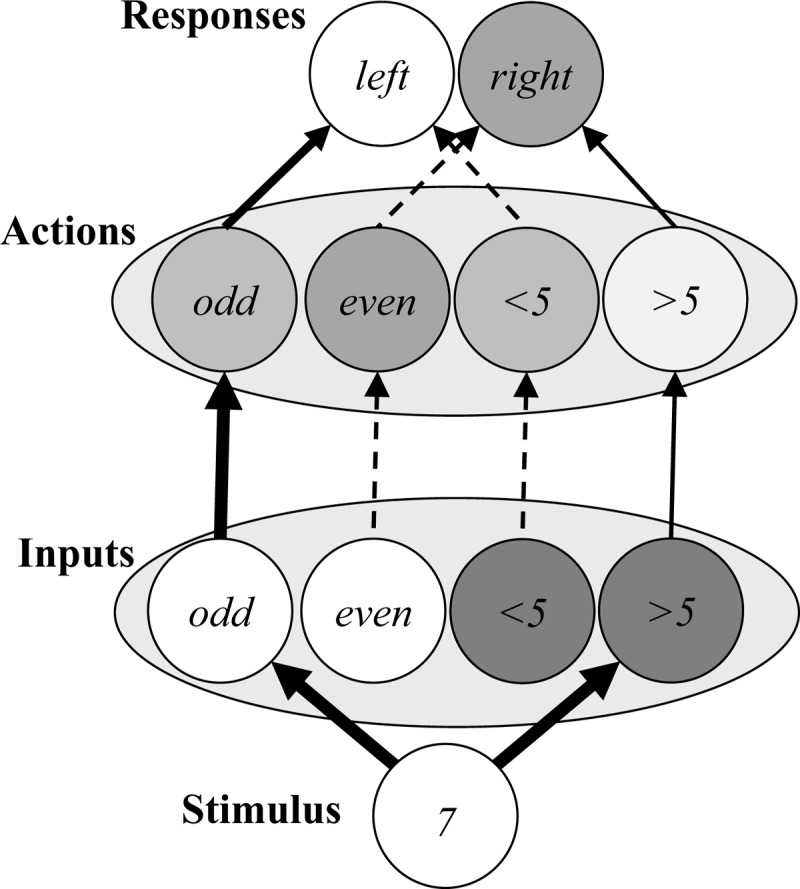
Simplified visual representation of the CARIS model as it applies to cued task switching. The inputs are filtered in favour of the current task (parity, white shading) and against the other task (dark shading). Actions are partially filtered on the basis of the prior task (in this example, magnitude) and also on the basis of whether the response on the prior trial was linked to the same or different action. The model contains a number of other parameters (not represented here) for fitting specific effects. *Notes*: brightness = filtering advantage for Actions and Inputs and activity level for Stimuli and Responses, arrow thickness = coactivation strength, dashed arrows = currently-irrelevant connections.

Next, consider the CARIS model of Meiran and colleagues (2008). CARIS is a computational model with a conglomerate of parameters for various individual effects, but perhaps the core component of the model is the filtering mechanism that assumes carryover filtering biases from the prior task set. Like the Gilbert and Shallice (2002) model, then, CARIS assigns an important role to task-set control in producing switch costs (unlike the PEP), though does make some extra provisions for other biases. Of all the alternative models to the PEP, the CARIS model fairs best, though this is a bit deceptive, because for many of these comparisons the model *must* fit the data (as explained below) and the resulting fit is out of spirit of the original model (also explained below). Note that the CARIS model can be reconfigured into various different versions depending on whether it is assumed that filtering for the current task (separately for inputs and actions) is determined by the goal of the present task versus carryover from the prior task. However, in the final selected model of Meiran short cue-target intervals were modeled assuming that filtering of the target is correctly based on the present task (e.g., parity inputs are biased over magnitude inputs when the current trial requires a parity decision), whereas filtering of the action passively carries over from the previous trial (e.g., there will be a filtering bias in favour of magnitude actions after a magnitude task). This is illustrated in Figure 11.

The CARIS model has various parameters that directly fit to certain effects, many of which are not illustrated in the figure. For instance, the model correctly produces a cue repetition benefit because it has a parameter that is fit to exactly match the observed benefit for cue repetition trials (called *D_SWITCH_* in the original paper) which is completely independent of the model as represented in the figure. The model also produces a response repetition benefit for task repetitions that is reversed for task switches, which is also explicitly modeled (*w_CR_*). Though not illustrated, the model also produces a task-rule congruency effect (modeled with *w_I_*). However, the model does not produce a complete repetition benefit for cue-RR or rep-RR trials (there is a small numerical difference, but only due to the fact that there are more congruent trials than in the cue-AR and rep-AR trials), and it also does not produce any differences in binding effects for task repetitions with versus without a cue repetition.

Most interestingly, the model appropriately produces a very small true switch cost. Because the CARIS model fits parameters to data like a regression, it is not surprising that the true switch cost comes out small in the fit, as this switch cost is small in the data that the model is being fit to. Interestingly, though, this switch cost results exclusively from the response repetition parameter (*w_CR_*) and the parameter that effectively fits the “true” switch cost, *w_A_*, is fit to having zero bias for the prior task (0.0, where 0.0 = no bias and 0.5 = complete carryover of previous response filtering). This is, of course, rather inconsistent with the spirit (and parameter; 0.014–0.040) of the original model and indicates that the model is effectively control free in this fit. That is, the original CARIS model did overattribute a much larger proportion of the switch cost to a true cost of switching (like the Gilbert & Shallice model) and asking the model to fit the switch cost decomposed into the different binding conditions leads to a drastically different final model. The model does not make clear error rate predictions, but one might reasonably suppose a similar ordering of means.

**Figure 12:**
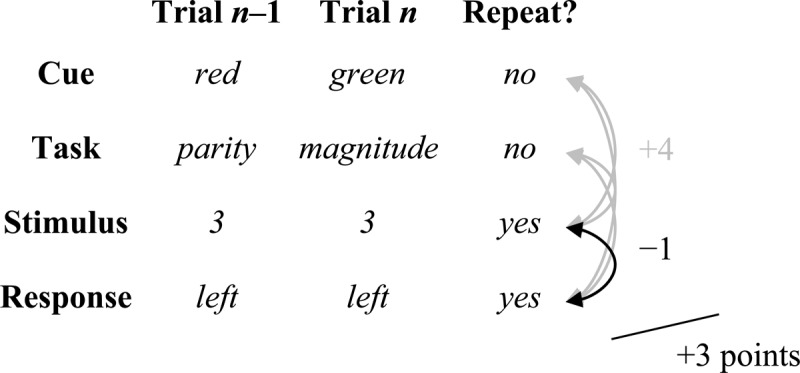
The Altmann model as it applies to cued task switching. Repeated features paired with another repeated feature count as –1 point, and repeated features paired with a non-repeated feature count as +1 point. There are no points for pairs of non-repeated features (e.g., the cue and task in this example). The example is of alt-RR trials, but each trial type is computed the same way.

Next, consider the Altmann (2011) mathematical model. This model has a similar “spirit” to the PEP model in that both models take into consideration binding biases. The Altmann model works with a “points” system, illustrated in Figure 12. The model attributes negative points for each repeated feature that is paired with another repeated feature (e.g., if the stimulus and response both repeat, this counts as 1 point) and positive points for each repeated feature that is paired with a non-repeated feature (e.g., a new response to a repeated stimulus counts as +1 point). Conditions with fewer points are expected to be faster than conditions with more points. Thus, the model largely predicts only ordinal differences between conditions. However, as mentioned earlier, these have been scaled to mean participant response time to facilitate comparison. The features considered included the cue, task (or goal), stimulus, and response, but not the decision. The simulated data are presented in Figure 10. Notably, the model fit is quite poor, with numerous qualitatively wrong predictions. For instance, the true switch cost is actually reversed (grey bars). Generally, the rudimentary point counting system makes some unintuitive predictions. For instance, repeating the stimulus and response on an alt-RR trial only counts as one negative point (speeding) for the stimulus-response match, but four positive points (slowing) because both the repeated stimulus and response mismatch both the task and task cue. The model also makes no considerations for task-rule congruency (not illustrated), which in the PEP model results from the same binding processes that produce better fit to the same 10 conditions. Thus, the Altmann model does capture some of the things that traditional task switching models miss out on almost entirely (i.e., it produces some binding effects), but has notable shortcomings in qualitative fit. Error rates are not presented, but the model should (presumably) predict the identical ordering of means for errors as for response times.

**Figure 13:**
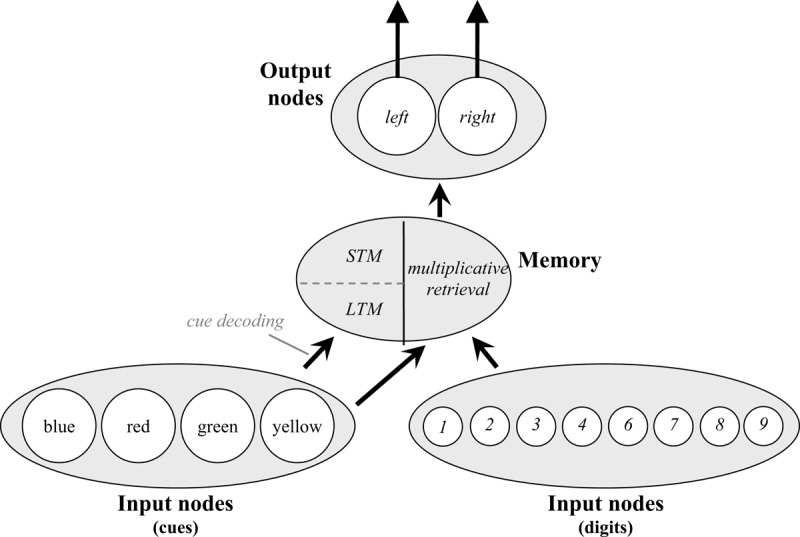
The Schneider and Logan (2005, 2009, 2014) model as it applies to cued task switching. The cue is initially decoded and is influenced both by priming of repeated cues in short-term memory (STM) along with semantic connections between cues in long-term memory (LTM). The cue and stimulus then multiplicatively determine retrieval of a response.

Finally, consider the Schneider and Logan (2005, 2009, 2014) priming model illustrated in Figure 13. Like the PEP and Altmann models, this model is heavily inspired by exemplar models of memory. We use the original 2005 version of the model, but the subsequent modifications are likely irrelevant for present purposes (the 2009 paper adds provisions for prime stimuli, and the 2014 paper introduces “background noise” activation of target inputs). The model assumes two primary things. First, on cue presentation, the cue is compared to memory to determine the appropriate task. A repeated cue can be processed more quickly than a non-repeated cue, and a related cue can be processed more quickly than an unrelated cue. Initially, this latter related-cue advantage was applied to the case where the two cues for a task are meaningful and semantically related (e.g., “odd” and “even” as cues for parity) and the benefit for processing a related cue (e.g., “odd” after “even”) was due to semantic priming. One might argue that the same might occur for meaningless (e.g., colour) cues if they become associated via their shared instructed link to one task (e.g., blue and red become related because they are both associated with the parity goal). Also to give this model the “benefit of the doubt” we allowed the model to make this assumption, effectively meaning that the model is equipped to perfectly match the overall differences between cue repetitions, task repetitions, and task alternations (like CARIS, this model primarily just sets parameters to equal observed effects). Second, once the stimulus is presented, the model searches through memory for the response associated with the (multiplicative) combination of the cue and stimulus. Strictly speaking, this is computed in a formula that weights activation of the correct response and the incorrect response, with the stimulus-linked response for the relevant task more strongly weighted than that for the relevant task (e.g., 7 activates odd to a greater extent than >5 on parity trials). The model does not consider bindings between stimuli, decisions, and responses. Note also that this model does not implement control of the type proposed by task-set reconfiguration and inertia accounts at all.

The results of this model are also presented in Figure 10. As can be observed, the model does produce the main effect differences between cue repetitions, task repetitions, and task switches. Though not illustrated, the model also produces a task-rule congruency effect. However, the model systematically fails to capture any of the remaining binding effects. The differences that are observed in the figure are exclusively due to the different relative frequencies of congruent and incongruent trials in each of the cells. The inability of this model to simulate binding effects (other than for cues) is not surprising, because the model simply has no provisions for modelling stimulus and response bindings. As with the Altmann (2011) mathematical model, this priming model shares some common underlying ideas with the PEP model on the contribution of binding biases to task switching phenomena. However, the PEP model goes further by implementing a neural network that considers a wider range of binding biases. It is perhaps also worth mentioning as an aside that the priming model is completely control free (or at least in the sense that it does not implement a task-set reconfiguration or inertia mechanism), as the difference between task repetitions and alternations (without a cue repetition) is due to inter-cue priming and not task-set control. If the inter-cue priming assumption discussed above is not made, the model will not produce the “true” switch cost. This is also a caveat to consider for the “true” switch cost observed in the PEP model and for the participant data itself: the remaining “true” switch cost may also be due to binding biases of a sort not implemented in the PEP, such as inter-cue priming.

### Summary

In this section, we aimed to illustrate how differently the PEP model produces switch costs relative to other models. Most notably, the PEP model produces switch costs as a result of feature integration (i.e., memory retrieval effects of recently-encoded events), with relatively less importance attributed to the switch in the task itself (i.e., there is “goal inertia,” but this only explains a part of the observed switch cost). Most traditional task switching models, however, attribute the switch cost (either exclusively or primarily) to a true cost of switching. Some accounts have considered exemplar-inspired notions, but the PEP framework is unique in its implementation of a memory store. The aim of this section was not to “discredit” other models, but rather to show that the PEP framework captures something unique. Further, this systematic variance between binding conditions that the PEP explains is worth considering in future modelling efforts as it can obscure what modellers are attempting to simulate. The most dramatic example of this is the CARIS simulation: asking the model to fit said variation between binding conditions effectively “broke” the control component of the model (i.e., the amount of variance attributed to control is exaggerated if not considering the binding contributions in the simulation).

## Simulation 6: Parameter Sensitivity

Showing that a model can fit well to a given pattern of data does not necessarily imply that the model is good. Especially as the number of parameters in a model increases, it could potentially be the case that a model could simply fit *whatever* pattern of results it is asked to model (Roberts & Pashler, 2000), in any direction. This is not by necessity true. Many model parameters, for instance, may simply be incapable of doing anything other than changing the rough scale of the results. For instance, a decay rate on node activations changes only the relative speed of selecting a response and not the direction in which a given effect is fit (i.e., positive *or* negative). Our subjective understanding of the PEP model is that it is not able to fit effects in any direction. For instance, the model is equipped to learn regularities, but it seems unlikely that the model would be *impaired* by high contingency events over low contingency events in any parameterization.^9^ Similarly, binding effects should have an a priori direction. Every model, of course, can be “broken” to the extent that it produces a null effect. As a simple example, any model of learning will produce a null learning effect with a learning rate of zero. Similarly, the magnitude of such a learning effect could easily vary with parameterization. Are, however, the qualitative predictions of the PEP model fixed? That is, does the model fit the observed data simply because it will fit any data, or because the conceptual considerations of the model imply a specific pattern?

We considered several potential approaches to testing our subjective notion that the PEP model has relatively fixed qualitative predictions and settled on an approach that seemed feasible suggested by Roberts and Pashler (2000): see whether the model is capable of producing reversed effects. Other approaches, such as testing the entire parameter space of the PEP model are largely impossible. The PEP has a sufficient number of parameters that it suffers from the “curse of dimensionality.” That is, there are far too many parameters to test all combinations across the entire orthogonal range of every parameter. Moreover, PEP simulations are far too computationally expensive (i.e., take too much time) to test large numbers of parameterized variants. An alternative approach, however, is simply to do the reverse of what we did to fit the model in the first place. That is, we can use an evolutionary algorithm to see whether the model is capable of fitting the reversed pattern of results. In particular, instead of asking the model to fit to the observed condition means, we asked the model to fit to the observed condition means subtracted from twice the mean of condition means (i.e., mean of means ∙ 2 – condition mean). Naturally, this means that the mean and variability across conditions remains the same as before, except that the model is asked to produce opposing predictions. Hypothetically, this could be done separately for each effect of interest (e.g., switch cost, task-rule congruency, etc.), but this test ensures that if the model is able to reverse *anything*, then it will be able to reduce error (i.e., relative to null predictions). In contrast, if the model simply does not have the ability to reverse its predictions (e.g., produce a cue repetition *cost* or task switch *benefit*), then the model should be able to do no “better” than null effects across the board.

Starting from our initially fit values from Simulation 1, we set a relatively moderate parameter mutation rate (.3) and mutation extremity (.3), started with a parent population of 40, and ran 1,000 children (see Appendix C on the evolutionary algorithm). In other words, the model tries random combinations of parameters, replacing those in the “population” that produce poor fit with those that improve fit. This is an extremely effective way of finding an improved parameterization (for a review, see Vikhar, 2016). That is, if there is a parameterization that allows the PEP model to reverse its predictions, this approach should find it. The initial parent population and all of the children were run with a sample size of 50 to speed simulation times. The final parent population was retested with 50 participants each (i.e., 2000 total) and we present the mean of these means. Table 2 lists all the parameters of the model that were allowed to vary in this sensitivity test (the same as those used in the original parameter fit). Some other parameters were fixed from the prior version of the model (or had a priori values, such as on/off values) and were not adjusted. All of the initially manipulated parameters in the table were allowed to freely vary (within their a priori range) in the evolutionary search.

**Table 2:** Original and newly-fitted parameters in sensitivity test with a priori range limits.

Parameter	Original	New Fit	A Priori Range

change (toward stimulus signal)	.034	.597	0–1 (proportion)
strength (target search)	3.4	.203	0–∞
strength (cue search)	1.14	.311	0–∞
noise (input)	.012	.252	0–∞
strength (decision search)	3.2	.405	0–∞
noise (decision)	.25	.323	0–∞
preparation (base)	.4	.282	0–∞
strength (response search)	3.5	.389	0–∞
noise (response)	.0026	.357	0–∞
decay (goal)	.007	.229	0–1 (proportion)
threshold (goal)	.1	.244	0–1 (activation range)
strength (goal)	1.37	.446	0–∞
noise (goal)	.66	.413	0–∞
weight (goal-decision)	.88	.289	0–∞
decay (input/decision/response)	.086	.397	0–1 (proportion)
decay (episode)	.0022	.290	0–1 (proportion)
loss (episode connection)	.045	.434	0–1 (proportion)
negative weights (episode)	–.11	–.36	–(0–∞)
restore (instruction episode)	.285	.218	0–1 (plausible range)
search (goal, after goal selected)	.1	.492	0–1 (proportion)
search (stimulus, after decision)	.95	.252	0–1 (proportion)
search (response coding)	4.0	.365	0–∞

**Figure 14:**
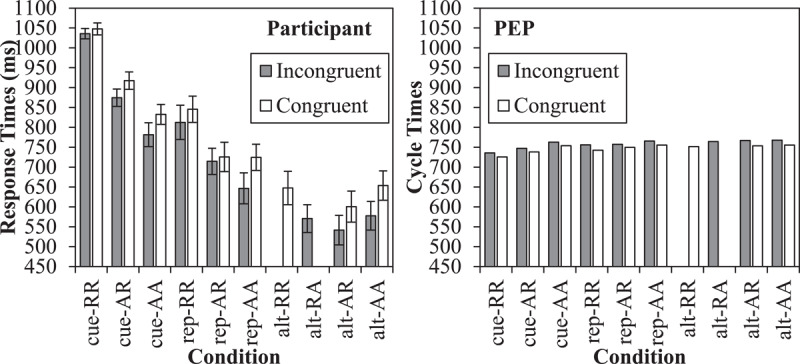
Parameter sensitivity test, with reverse-direction original response times (with standard errors) and Simulation 3 cycle times from the PEP. Notably, the PEP model is unable to fit reverse-direction effects.

The results of the evolutionary search are presented in Figure 14. Here, we have also split up the 10 feature integration conditions as a function of task-rule congruency (note that alt-RR can only be congruent and alt-RA can only be incongruent), which was also how we fit the initial model in Simulation 1. As anticipated, the model was unable to reverse any key predictions. Instead, and also as predicted, the model was only able reduce the differences between conditions to near (but not complete) zero. Errors are not presented for brevity, but produce the identical pattern. Thus, although the PEP model may be a large framework with many parameters built in, the model does not have much maneuverability in what it can predict. Indeed, most of the parameters (both those we fit in the evolutionary search and those we did not) are merely “information transmission speed” parameters and do not change the fundamental processing rules of the system.

## General Discussion

In the present work, we used the Parallel Episodic Processing (PEP) model to simulate a range of findings from the task switching domain. In Simulation 1, we observed that feature integration biases systematically inflated the switch cost, as in the participant data (Schmidt & Liefooghe, 2016). These biases come from several interrelated sources. On task repetitions, all binding biases (i.e., repetition of the cue, stimulus, or response) *facilitated* performance relative to the “complete alternation” condition (rep-AA). Cue repetitions lead to quick retrieval of the just-encoded episode, leading to quick determination of the goal. This further boosts stimulus-response binding biases (cue-AR and cue-RR), because the stimulus and response are also primed by retrieval of the just-encoded episode. Similarly, response repetitions are especially fast given that the same response (e.g., left) is made to the same decision (e.g., odd) as what was just encoded into memory (see also Longman et al., 2018, for more on the importance of decision-response bindings), and this retrieval benefit is even larger if the stimulus repeats, too.

On task alternations, however, cue repetition benefits are impossible and retrieval of the just-encoded episode is less consistently desirable. Repetition of the stimulus only (alt-RA) leads to a *cost*, because the stimulus was just linked to a *different* decision and a *different* response on the preceding trial. Similarly, a repeated response on alt-AR trials requires making the same response (e.g., left) to a *different* decision (e.g., “odd” vs. “<5”). When both the stimulus and response are repeated on a task alternation (alt-RR), the situation is more complex, given that the stimulus-key link is consistent with the previous trial, but the stimulus-decision and decision-response connections are inconsistent. Thus, whether this condition comes out faster or slower than a complete alternation (alt-AA) could be highly parameter specific (unlike almost everything else in the PEP model), which also seems consistent with behavioural results (e.g., Akçay & Hazeltine, 2007; Notebaert & Soetens, 2003; Schmidt & De Houwer, 2011). Combined with the priming benefits for task repetitions, these binding costs for task alternations result in a substantial overestimation of the cost of switching the task itself.

As in the participant data, the PEP model did produce a remaining switch cost (i.e., rep-AA vs. alt-AA), at least in cycle times. This results largely because of residual activation of the previous task goal, which we might call “goal inertia,” as we also demonstrated in Simulation 1. In other words, determination of the current-trial goal can proceed more quickly on a task repetition. This “carryover priming” is consistent with some views of the origin of the switch cost (Allport et al., 1994; Allport & Wylie, 2000; Gilbert & Shallice, 2002), especially task-set inertia, but it is notable that this priming only explains a portion of the overall switch cost in the current model. Most of what the PEP produces is instead due to binding biases.

The PEP model also recovers several important, or “benchmark,” findings in the task switching domain. As already mentioned, cue repetition benefits are recovered by the model and this explains the largest portion of the overall switch cost. We also demonstrated that the model produces a response repetition asymmetry. In particular, the response repetition benefit is large on a task repetition, but not on a task alternation. In the PEP model, this is not (in any direct way) related to the switching of the task itself. Instead, it is precisely because a physical keypress response is linked to the same classification/decision twice in a row on a task repetition (e.g., “odd”-left → “odd”-left) that a large response repetition benefit is observed, whereas this is not the case on a task alternation (e.g., “odd”-left → “<5”-left), where there is an inconsistency in the decision-response binding.

We further demonstrated that the PEP model produces a task-rule congruency effect during cued task switching. In particular, incongruent stimuli (which require different keypress responses in each task) are responded to slower and less accurately than congruent stimuli (which require the same response in both tasks). This indicates that, though the system is able to determine the correct response in both tasks, competition (or crosstalk) between the differing task rules for a single stimulus can occur during memory retrieval, which slows down incongruent responses. Thus, the task-rule congruency effect is yet another logical consequence of the binding of stimuli to responses in memory. Indeed, unlike the other models considered here, the task-rule congruency effect is not produced by a different mechanism than other effects: the exact same learning process that produces more transient “feature integration” biases also produces longer-term task-rule congruency effects (and contingency learning and skill acquisition; see Schmidt et al., 2016). The simulation also produced an asymmetry between the switch cost and task-rule congruency, with larger switch costs to incongruent stimuli. This is consistent with some reports (including the participant data that we simulated) but is not always observed. Like the switch cost itself, this interaction was due to confounding feature integration biases.

### PEP and task-set control

Switch costs are often attributed to a reconfiguration of task-sets (Meiran, 1996; Rogers & Monsell, 1995) or task-set inertia (Allport et al., 1994). In our model, the majority of the switch cost is explained by simple feature binding: stimuli bound to responses, cues bound to goals, decisions bound to responses, etc. This deviates from most views, which assume that the switch in the task-set itself entails a major cost. However, might the remaining switch cost be explained by one (or more) of these task-set control processes? Leaving aside the question of whether task-set control actually induces effects in task switching, we consider whether the PEP model itself is consistent with any of these accounts. In prefacing this discussion, we note that each reader might interpret concepts like “task-sets,” “task-set reconfiguration,” or “task-set inertia” differently. As such, some of these notions might be considered more consistent with the current instantiation of the PEP model if interpreted differently than the standard views that we describe below.

First, what could be considered as task-set control in the PEP model is very minimal compared to other models. The Goal nodes, along with their connectivity to other (e.g., cue Input and Decision) nodes, might be argued to be a computational implementation of task-sets, but all these nodes do is retrieve the viable decision options it is considering (i.e., “odd or even?” or “more or less than five?”). Calling these nodes “task-sets” may, in our view, be overstating their function. By another view, it might be proposed that the binding processes implemented as a whole in the PEP are the nuts and bolts of task-set control. Depending on how one is willing to define “control” and “task-set,” this view might be complicated by the bottom-up nature of the priming in the model. Similarly, speaking in terms like “processing pathways” seems to imply different outcomes than speaking in terms of bindings. For instance, a “task-set” priming the “stimulus translation rules” of the parity set would seem to imply favouring of all stimulus-response correspondences in the set indiscriminately, rather than producing especially large benefits for just-encountered stimulus pairs. Thus, if the PEP model is to be viewed as an implementation of task-set control, our implementation also suggests some very different ways in which to conceptualize this control and what implications it should have for performance.

Even ignoring this caveat, it seems clear that the PEP model is not consistent with the task-set reconfiguration account. According to this account, reconfiguration of the task-set on task alternations is a discrete process that must occur before one can continue on with classifying the stimulus appropriately and deciding on a response, which definitely does not occur in the PEP model. Similarly, the reconfiguration view seemingly implies (in some models explicitly) that there is a “switch detector” mechanism to determine whether or not the task-set must be reconfigured. A model by Brown, Reynolds, and Braver (2007) relies on a related “cognitive control loop” to make this detection and produce switch costs. This is unlike the PEP framework, however, which does not “watch” for switches and, indeed, does not do anything inherently different on task repetitions and alternations. Instead, on each and every trial the model sees the cue and searches through memory for what it must do. Thus, it might be argued that the model “configures” itself on every trial, only faster in one condition than the other (see also, Logan & Bundesen, 2003), but this is typically not what researchers mean by a “reconfiguration cost.” Furthermore, the overall switch cost is decidedly *not* a direct measure of reconfiguration in the PEP.^10^

Next, consider the task-set inertia account (Allport et al., 1994; Allport & Wylie, 2000; Gilbert & Shallice, 2002; Meiran et al., 2008). The task-set inertia account also assumes competition between past and previous task-sets and/or priming of repeated tasks (Yeung & Monsell, 2003a, 2003b), but not as a discrete step. That is, the task-set in the previous trial has not completely decayed by the time the next trial begins (or is involuntarily re-retrieved) and this causes interference on a task alternation. As the PEP model is currently programmed, there is some residual activation of the previous-trial goal by the time the next trial begins (in part reinforced by recurrent episodic retrieval) and we demonstrated in Simulation 1 that this “goal inertia” does explain the “true” switch cost in PEP-simulated data. Thus, the model is at least partially consistent with task-set inertia models (e.g., Gilbert & Shallice, 2002). Crucially, of course, the PEP model ascribes substantially less of the switch cost to carryover activation of the goal. Put differently, the effect of “inertia” of the prior goal is much weaker in the PEP model.

Another question relates to the role of working memory in task-rule congruency effects. As mentioned earlier, there has been debate over the role of working memory versus long-term memory in producing the task-rule congruency effect. While one view proposes that conflict between task rules occurs within working memory (Meiran, 2000), another view proposes that only the relevant task rules occupy working memory, and task-irrelevant rules influence performance via retrieval from long-term memory (Hommel & Eglau, 2002; Kiesel et al., 2007; Mayr & Kliegl, 2000; Meiran & Kessler, 2008). In the PEP model, the task-rule congruency effect is produced exclusively via retrieval from long-term memory. The currently active goal does assure stronger retrieval of task-relevant memories than task-irrelevant memories. Whether this counts as “working memory” we are unsure (and may be consistent with the notion that the task-rule congruency effect emerges from an interaction between a mediated and non-mediated route; see Schneider, 2015), but it is noteworthy that incongruent trial slowing in the model is due to slower retrieval of the task-appropriate decision and response from long-term memory. Thus, while all accounts of task-rule congruency effects, in one way or another, assume some form of “crosstalk” between the stimulus-response bindings for the two tasks, the current implementation places a much larger role on long-term memory retrieval than most prior views.

As an added thought, task-sets have been previously proposed to be encoded along with stimulus information in episodic memory (Allport & Wylie, 2000; Koch & Allport, 2006; Waszak et al., 2003), and retrieved to influence responding on subsequent trials with similar stimuli. Leaving aside the previous discussion as to whether Goal nodes constitute task-sets, the use of Goal nodes in the PEP model could be regarded as a computational instantiation of this notion. Indeed, Goal nodes are encoded into episodic memories, along with the cues, target stimuli, responses, and internal decisions. Furthermore, the ability of the model to continue to do the correct task in response to a given cue is dependent on the continued binding of cues to goals. More broadly, there has been increasing interest in the notion that “simple” learning/binding biases and more abstract “control” biases might be falsely dichotomized. In particular, some have considered the notion that control is simply part of learning, only instead of learning between concrete stimulus and response features, control reflects the same learning between more abstract features like attentional settings or control parameters (e.g., see reviews by Abrahamse, Braem, Notebaert, & Verguts, 2016; Braem & Egner, 2018; Egner, 2008). The PEP model offers a formalization of this idea.

It is also worth explicitly noting that an important aim of the present manuscript was to demonstrate how many key findings normally interpreted in terms of task-set control may instead be due to simpler binding biases. This is not to argue, however, that there is no room left for such control mechanisms to account for other aspects of task switching performance. One example is strategic preparatory effects. For example, Monsell and Mizon (2006; see also, Mayr, Kuhns, & Rieter, 2013) observed that the size of switch costs and preparatory costs is modulated by switch expectancy. In particular, effects are much larger when switches are rare and smaller when switches are frequent (intermediate with chance frequencies). Intentionally preparing for the just-executed (or opposite) task when the task contingencies indicate that a task repetition (or alternation) is likely is certainly something that the present version of the PEP model does not implement. Although in principle such effects might (at least in part) be modelled as contingency learning at the goal level (e.g., parity primes parity with frequent repetitions or magnitude with frequent switches), such effects might also indicate a top-down control influence. There are many other examples of potential top-down expectancy influences, such as in voluntary switching paradigms (e.g., Arrington & Logan, 2004b; for a review, see Arrington, Reiman, & Weaver, 2015) and the difference in position-in-run effects between predictable and unpredictable switches (Monsell, Sumner, & Waters, 2003).

### Other models of switch costs

In Simulations 2–5, we compared the PEP model to other computational models of task switching. Here, we summarize these differences and consider yet other models of task switching. The Gilbert and Shallice (2002) model (also a dynamic neural network) implements the typical task-set inertia notion, relying almost exclusively on carryover of task-set activations from the previous trial to produce the switch cost. That is, the just-executed task node is partially active at the beginning of the next trial and the not-executed task node is partially suppressed. Although the PEP model involves some degree of carryover like this as well, the Gilbert and Shallice model attributes the entirety of the switch cost to this carryover. In contrast, in the PEP model some “task-set inertia” exists (i.e., there is some bias for a repeated goal), but this bias is rather small and most of what the PEP model produces (including the switch cost) is due to the binding biases discussed earlier. As such, the two models have some surface similarities, but the PEP model produces a switch cost in a very different way.

The CARIS model (Meiran et al., 2008) produced a reasonably good fit to some findings, including the cue repetition benefit, task switch cost (sort of), task-rule congruency effect, and response repetition asymmetry. All of these are rather directly modelled by means of regressors that fit the individual effects, and the model fails to account for other binding biases (e.g., stimulus-response). More “inconvenient” for this model, however, is that the fit of the CARIS model to the data of Schmidt and Liefooghe (2016) was inconsistent with how the model is supposed to work in concept: the model did not fit a “true” switch cost (there was a difference for the key switch cost contrast due to the explicit modelling of the response repetition asymmetry, but no carryover of the previous task-set). That is, the CARIS model is supposed to produce a “true” switch cost via control over task-sets (like the model of Gilbert & Shallice, 2002), but did not.

The Altmann (2011) and Schneider and Logan (2005, 2009, 2014) models are similar in concept (though not in formalisation) to the PEP model in that they are similarly inspired by the episodic modelling tradition. The Schneider and Logan model successfully produces cue repetition effects, switch costs, and congruency effects, though it does not have any previsions for other types of binding biases (e.g., stimulus-response). The Altmann mathematical model codes for many of these binding biases (although not related to the decision), but the point counting system makes some unintuitive (and incorrect) predictions. The Altmann model also fails to account for task-rule congruency effects and the switch cost proper. Note that an earlier version of this model from Altmann and Gray (2008) was based on the ACT-R production framework (Anderson, 2007; Anderson et al., 2004) and, like the PEP, included stimuli, cues, tasks (goals), decisions, and responses. It also included episodic traces. However, these traces only coded for the task. Thus, unlike the subsequent model by Altmann (2011), it did not account for feature integration effects. Relative to these other exemplar-inspired models, the PEP framework is more dynamic (i.e., a neural network) and feature integration, congruency, and other effects emerge naturally out of simple memory storage and retrieval processes – the same processes required for instruction implementation and learning. Thus, we believe that the PEP framework is a step forwards, though a step in a similar direction. Stated differently, the PEP model is a more fleshed out implementation of the notion that episodic biases combine to produce task switching effects than prior implementations.

There are many other, much more complex models of task switching. For instance, a model by Brown and colleagues (2007), discussed earlier, includes multiple cognitive control loops. The switch cost results from a direct monitoring of task conflict (i.e., an explicit comparison between the task on the current and previous trial), which triggers top-down control to suppress the task-irrelevant goal and boosts the task relevant goal if a conflict (task switch) is detected. This is an extension of conflict monitoring theory (see Botvinick, Braver, Barch, Carter, & Cohen, 2001). Similar to task-set reconfiguration or inertia accounts, it is assumed that a cost is acquired when switching tasks, but it is additionally assumed that there is a task conflict monitor that has to detect whether a reconfiguration is required. We did not implement this model as it (like Gilbert & Shallice, 2002) does not have provisions for dealing with feature integration biases.

A related paper by Sexton and Cooper (2017) applied a variant of the Gilbert and Shallice (2002) model, but also included a task conflict monitor. When the task conflict monitor detected two active task nodes, both were inhibited. Thus, activation of the immediately-preceding task does carry over to the next trial (producing facilitation on task repetitions and costs on task alternations), but the previous task node is inhibited in the process. This task-node specific inhibition carries over for a longer period of time. Via this mechanism, when a third trial begins, the first task (*n*–2) is still receiving inhibition. This predicts (albeit, depending on parameterization) that performance will be *impaired* when the task on Trial *n*–2 repeats on Trial *n*. This finding is termed *n*2 *task repetition costs* (Koch, Gade, & Philipp, 2004; Mayr & Keele, 2000; for a review, see Koch, Gade, Schuch, & Philipp, 2010). This long-lasting inhibitory mechanism (i.e., inhibition of recently-encountered tasks/goals that are no longer appropriate) may not be as directly relevant to the current simulations of immediate-trial switch costs and feature repetition effects. That is, the long-lasting inhibition is more applicable to the impact of more distal effects, so we did not reproduce this model, either. However, the *n*–2 *task repetition cost*, more generally, is an interesting phenomenon and it seems unlikely that the current instantiation of the PEP model would be able to account for it. There is some evidence that simple *n*2 episodic binding biases contribute to *n*–2 task repetition costs (Grange, Kowalczyk, & O’Loughlin, 2017), but this does not seem to account for the whole effect. Two possible adaptations might be possible to model *n*–2 task repetition costs. First and most obviously, one could instantiate a similar mechanism to Sexton and Cooper. Whether an explicit conflict monitor would be necessary is uncertain, but goal competition (which already occurs in the PEP) could be programmed to have lasting influences (i.e., beyond the immediate moment when two Goal nodes are concurrently active). Another possibility would be to assume either (a) the need to inhibit Goal X is coded into the newly-formed episode and this inhibition gets re-retrieved on the following trial (similar to the “do not respond” tags in the episodic account of negative priming by Neill, 1997), or (b) episodes themselves get temporarily inhibited when they have pointed us in the wrong direction.

One other limitation with the current simulations is that we focused on one particular version of the task switching paradigm. Although the Schmidt and Liefooghe (2016) cued task switching design is relatively prototypical and although we analysed a great deal of complexity within this one task (e.g., the binding decomposition), further work could be done to apply the model to other task switching scenarios. One particular domain of interest (including in past modelling efforts) is the modulation of the switch cost as a function of different time intervals. For instance, it has been observed that the switch cost decreases as the cue-target interval increases (e.g., Meiran, Chorev, & Sapir, 2000). Manipulations of cue-target intervals have frequently figured into task switching debates, for instance, on the relative merits of task-set reconfiguration and task-set inertia views (Meiran, 1996; Monsell & Mizon, 2006). In the present work, we only simulated one cue-target interval. Relatedly, increasing the intertrial interval (i.e., the time between the end of one trial and the cue for the next) also decreases the switch cost, even with a fixed cue-target interval (Meiran et al., 2000). Also important is the *residual switch cost*, which is the finding that the switch cost is not completely eliminated with advanced preparation (for an exception, see Verbruggen, Liefooghe, Vandierendonck, & Demanet, 2007; but see Schneider, 2016). According to some views, the switch cost is never entirely eliminated with advanced preparation because there is a stage of task preparation (e.g., activation of task rules) that waits until the target stimulus is presented (Rogers & Monsell, 1995; Rubinstein et al., 2001). Another view proposes that the currently-irrelevant task-set is inhibited during a trial, and this inhibition persists across trials, thereby making it harder to execute a just-inhibited task (Schuch & Koch, 2003). Yet other views propose persistent activation of previous task-sets across longer periods of time (Allport et al., 1994) or re-retrieval of task-sets on stimulus presentation based on stimulus-task-set bindings (Waszak et al., 2003, 2004).

Globally, persisting switch costs with advanced preparation are one of the most significant findings discussed in the task switching literature, which we have not explored here. This obviously represents a limitation with the present work. Further work might therefore investigate whether the PEP model can also match the time-course of such effects. The PEP model might already be able to explain reduced switch costs with delays, because the earlier resolution of the task-appropriate goal (with a long CTI) might reduce any “head start” advantages for repeated cues, and therefore result in a reduced impact of memories linked to the inappropriate goal (e.g., the prior-task goal on a task switch). However, there may be more to preparation effects than simple decay and whether a residual switch cost remains in the model (as it should) remains to be seen.

Indeed, also past models have fallen short in simulations of interval effects. For example, larger switch costs with shorter response-cue intervals (RCIs) is *only* observed when short and long intervals are intermixed, not when manipulated between participants (Altmann, 2005; Koch, 2001). Horoufchin, Philipp, and Koch (2011a, 2011b) similarly found the same difference between intermixed and blocked RCIs (but see Monsell & Mizon, 2006). Related findings have been observed with CTI manipulations: robust CTI effects with intermixed intervals, no effects with between-participant manipulations, and carryover effects with blocked CTIs (Altmann, 2004). These findings are not captured by extant models. Horoufchin, Philipp, and Koch (2011a, 2011b) interpreted such findings in terms of episodic biases (related to the *temporal distinctiveness* of memories; e.g., see G. D. A. Brown, Neath, & Chater, 2007). Thus, the PEP framework might be particularly useful for exploring some of these notions computationally. For example, “temporal distinctiveness” might be implemented by coding temporal context as an input to the model.

Independent of this, the present feature integration decomposition of task switching also suggests an interesting new question: Is there a residual switch cost due to limits on advanced preparation of the *task* (Mayr & Kliegl, 2000, 2003; Rogers & Monsell, 1995; Rubinstein et al., 2001) as typically proposed or is the residual switch cost due to residual *feature integration* biases? In other words, it may be the case that advanced preparation does eliminate the true cost of switching the task goal, but this is concealed by remaining feature integration biases. Findings from the binding domain do show that stimulus-response binding effects are reduced with increased delays (Frings, 2011; Moeller & Frings, 2017), but whether the residual switch cost is due to residual binding or residual switch costs is unclear given the failure to control for feature integration biases in past reports with interval manipulations. It might be the case that advanced preparation for a task switch is not as limited as previously assumed. Future empirical research might explore these possibilities.

As in the *n*–2 repetition costs and interval manipulation examples above, there are undoubtedly many ways in which the PEP account given here is incomplete. Limitations aside, the elegance of the PEP model is that it captures complexity with simplicity. It merely stores what it has experienced (e.g., the stimuli that it saw, the response it made, the goal it had, etc.) and solves a broad range of tasks by simply retrieving these memories. Similarly, nothing in the model is paradigm specific. The model can simulate a range of task switching observations for the same reason that it can remember and implement task instructions and goals, learn contingencies, improve with practice, and so on (e.g., see Schmidt et al., 2016). Indeed, the PEP model is a general framework that can model task switching phenomena, rather than a model of task switching per se.

### Future directions

As mentioned earlier, the switch costs (and task-rule congruency effects) observed in the current report result from memory retrieval. In particular, these effects emerge as a result of the binding of cues to goals (e.g., Arrington & Logan, 2005; Logan & Bundesen, 2003, 2004; Logan et al., 2007), stimuli and decisions to responses (e.g., Goschke, 2000; Kleinsorge & Heuer, 1999; Kleinsorge et al., 2004), etc. in memory. Each of these biases have been considered before in the literature separately, but the PEP instantiation here novelly integrates these binding effects coherently in one framework. Further expansions on the idea that switch costs emerge via episodic retrieval are possible within the current framework. For instance, we already mentioned that our instantiation might suggest that residual switch costs are just feature integration effects in disguise (e.g., if what is left over after longer delays is due to binding biases). Research might therefore aim to study less-confounded switch costs (e.g., as in Schmidt & Liefooghe, 2016) with longer intervals, to see whether the cost of switching tasks is really as resistant to advanced preparation as assumed in the past.

Globally, a more systematic approach to dealing with binding influences in task switching is warranted in the future. Other than removing feature repetitions after the fact (as in Schmidt & Liefooghe, 2016), there are other potential approaches that have been used in the past (though typically not with complete controls) that could be considered. For instance, many experiments have included multiple cues per task and have disallowed immediate cue repetitions by design (e.g., Elchlepp, Lavric, & Monsell, 2015; Monsell & Mizon, 2006), used large stimulus sets where repetitions of individual stimuli are either non-existent or only occur at longer lags (e.g., Arrington & Logan, 2004a; Elchlepp et al., 2015; Monsell & Mizon, 2006), or increased the number of response alternatives such that response repetitions are rare (e.g., Waszak et al., 2003, 2004; Waszak, Hommel, & Allport, 2005). Switch costs have been observed with such controls, though complete controls for all binding biases in a single report with such methods has not been done previously. In any case, such approaches provide some alternatives over the approach of allowing all binding biases to start with, then deleting the majority of observations afterwards to obtain a “pure” measure of switching the task itself.

As also mentioned, the present model did simulate part of the switch cost as “goal inertia.” However, other views exist. For instance, in the version of the Schneider and Logan (2005, 2009, 2014) model that we simulated participants are said to first process the cue for its identity, then afterwards use the cue and stimulus in combination to predict the response (i.e., after learning cue-stimulus-response combinations). A “true” switch cost in this model is actually only due to inter-cue priming: slower processing of cues associated with an alternated task than with the repeated task. Combined with the cue-target response search, this notion does not require any control. That is, even when comparing task repetitions and alternations where no cues, stimuli, decisions, or responses repeat, an indirect binding effect might still benefit task repetitions. This could be the case if, for instance, the two cues (e.g., blue and red) for each task become associated (cue equivalence; see Honey & Ward-Robinson, 2002) via their shared connection to the same goal (e.g., parity) and/or to the same decisions (e.g., odd and even). The original models included inter-cue priming because the two cues for each task were semantically related (e.g., “odd” and “even” as primes for parity), but arbitrary cues could also become linked indirectly. Indeed, there is research outside of the task switching domain showing that mediated learning like this does occur (sometimes referred to as intersecting regularities; see Hughes, De Houwer, & Perugini, 2016; Liefooghe, Hughes, Schmidt, & De Houwer, 2020).

Linked to this notion, the cue-stimulus search in the Schneider and Logan (2005, 2009, 2014) model does not require task representations or goals. The authors do appeal to the notion of a “mediator” (Schneider & Logan, 2007) representation of the task (e.g., “parity”) in later work, which could be seen as similar to the goal priming in the PEP, except that the PEP also assumes representation of the decisions. In any case, participants can be instructed to perform in a manner that seems consistent with cue-stimulus-response learning, but do not seem to do this (exclusively) by default (Forrest, Monsell, & McLaren, 2014), whereas simpler animals like pigeons seem to learn via cue-stimulus-response learning exclusively (Meier, Lea, & McLaren, 2016).

The current simulations also, rather accidentally, suggest a potential resolution to an apparent inconsistency in past work on stimulus-response binding. In the original work by Hommel (1998), both complete (stimulus + response) repetitions and complete alternations (neither the stimulus nor response repeats) were found to be fast, whereas partial repetitions (i.e., repetition of the stimulus or response, but not both) were slow. It was suggested that partial repetitions entail a cost because a repeated stimulus must be rebound to a new response, or vice versa. In contrast, with both a complete repetition and complete alternation, no rebinding is needed. In the participant and simulated data, the feature integration biases for *task alternations* are consistent with this pattern (see Figures 3 and 4). However, other investigations suggest a different pattern, particularly with distracter-response binding procedures (e.g., Frings et al., 2007) and in the attentional control domain (e.g., Schmidt & De Houwer, 2011; Schmidt et al., 2014). In particular, complete alternations are typically the *slowest* trial type, with repetition of the response leading to an overall priming benefit, even if the stimulus does not repeat (i.e., partial repetition benefit). In the simulated and participant data in the present report the data is consistent with this overall response repetition benefit for *task repetitions*.

The current simulation results suggest a resolution to this conundrum. The PEP model *does* produce an overall response repetition benefit (magnified further by a repetition of the stimulus), as clearly observed for task repetitions. On a task alternation, however, there is also a *cost* associated with making a repeated response to a *different* decision (e.g., left key to “odd” then to “<5”) and a different decision to the same stimulus (e.g., “odd” then “<5” to 3). This works against any (overall) response repetition (and complete repetition) benefit. Notably, in the paradigm of Hommel (1998), a different decision is also made to repeated responses (i.e., based on a response cue for the prime trial and based on stimulus identity for the probe trial). Thus, “partial repetition costs” on switches are not due to “unbinding,” but to a mix of response repetition benefits and stimulus-decision and decision-response binding costs, which also explains why the pattern is completely different for task repetitions. Further work aimed at distinguishing between stimulus-decision, decision-response, and stimulus-response bindings (for related work, see Horner & Henson, 2011; Longman et al., 2018; Moutsopoulou, Yang, Desantis, & Waszak, 2015; Pfeuffer, Moutsopoulou, Pfister, Waszak, & Kiesel, 2017) might therefore aim to test this coherent binding account implied by the PEP model.

As another note, the current modelling results show that feature integration effects can emerge from a single long-term memory store (see also, Schmidt et al., 2016). Simulation of the relatively complex pattern of feature integration effects did not require special short-term memory event files that are separate from a long-term memory learning store, as has been previously proposed (Colzato, Raffone, & Hommel, 2006). In the PEP model, feature integration effects are a consequence of the same memory storage and retrieval processes that are required for learning, skill acquisition, and instruction maintenance. Though more parsimonious, further research might be directed at determining whether learning and binding effects are as intricately related as the current framework suggests (e.g., for some potentially problematic data, see Moeller & Frings, 2017).

Future modelling (or experimental) work might explore yet other “episodic” biases not yet implemented in the PEP model. Two examples discussed already above are temporal distinctiveness and inter-cue priming effects. One possibility is that the switch costs that remain after controlling for the binding confounds explored in the present manuscript are also explainable by binding biases. For instance, rep-AA trials might still be faster than alt-AA not because of a task-set reconfiguration or inertia, but because the non-repeated cues on rep-AA prime each other due to their shared overlap with the same goal.

The present work also suggests future potential applications in task switching and neighboring domains. One key notion in the PEP model is that performance is heavily impacted by similarity in inputs between prior and current events. In addition to item-specific triggering of task goals via stimulus-task bindings (Waszak et al., 2003, 2004), this might suggest that any change in similarity between stimuli, even with task-irrelevant contextual stimuli (e.g., the location a target happens to be presented), could modulate the binding effects that manifest as a “switch cost.” There are analogous results in the binding literature. For instance, Frings, Koch, and Moeller (2017) showed that stimulus-response binding effects are diminished when there is a stimulus selection context change. For instance, the complete repetition benefit of a triangle target and square distracter was smaller if the target object colour switched from light to dark blue and vice versa for the distracter object colour relative to when the colour context repeated. A similar notion might be extended to task switching.

The present manuscript also focused exclusively on behavioural effects in response times and errors, but there might be interesting extensions to other methods. For instance, Yeung, Nystrom, Aronson, and Cohen (2006) conducted a task switching experiment with fMRI and observed that brain regions associated with the currently-task-irrelevant stimulus (right fusiform gyrus for faces and left inferior temporal gyrus for words) was greater after a task switch than after a task repetition. They took this as evidence for carryover of the prior task (inertia). This interpretation may be correct and could be regarded as consistent with the goal carryover in the PEP model. However, while this study did avoid direct stimulus repetitions, decision-response bindings were not controlled. It may therefore be interesting to reinvestigate these issues in imaging studies with controls for binding biases. Similar concerns exist in ERP studies on cued task switching (e.g., Lavric, Mizon, & Monsell, 2008), where binding biases were also not controlled.

Similarly, it might be worth considering the implications of the present work on eye tracking. For instance, Longman, Lavric, Munteanu, and Monsell (2014) observed attentional biases toward previous task-locked stimulus locations in cued task switching with eye tracking. That is, participants had a bias to re-attend the stimulus location for the previously-executed task even on task switches where a new stimulus location had to be attended. Furthermore, participants were slower to attend the correct location on switch trials. Of course, this particular study involved spatial attention, which is different than the studies considered in the present report, but the results do seem consistent with a cost in task preparation for task switches. Also, task repetitions and alternations differ in this case not only in the task itself, but also in whether the eye movement response repeats (a repeated decision-response binding for the *task*) or alternates (a new decision and response) from the previous trial.

### Summary

Although switch costs are often discussed in terms of higher-order control processes, such as proactive or reactive control (Meiran et al., 2008), task-set inertia (Allport et al., 1994), task-set reconfiguration (Meiran, 1996; Rogers & Monsell, 1995), or cognitive control loops (J. W. Brown et al., 2007), the cost can also, at least in part, be understood in terms of feature integration effects, as illustrated with the current modelling effort. More globally, we hope that this paper, along with our other work with the PEP framework (Schmidt, 2013a, 2013b, 2016a, 2016b, 2018; Schmidt et al., 2016; Schmidt & Weissman, 2016) serves to illustrate how an episodic framework provides a coherent and unifying account of behaviour across a range of subfields of cognition. Indeed, this work aims to show how a great deal of complexity can simply “fall out” of some basic assumptions about memory storage and retrieval. Of course, it is unlikely to be the case that all findings within the task switching domain can be explained by simple episodic biases, especially given the rich variety of tasks and task manipulations explored in the domain. Indeed, it is not our aim to argue that cognitive control is irrelevant for behaviour generally or even for switching between tasks more specifically. In fact, some elements of the current implementation of the PEP model might even be viewed as implementations of control (e.g., goal retrieval) and these “control” processes might account for at least part of switch costs (albeit much less so than in traditional accounts). However, the more critical point is that the present work illustrates just how difficult it can be to study higher-order control processes. If one means to study something other than simple binding biases (e.g., task-set inertia), then one must be extremely careful when designing experiments and analysing data to rule out lower-level feature integration biases. Indeed, it is not even clear whether the remaining “pure” switch costs observed in studies like Schmidt and Liefooghe (2016) are due to control processes (e.g., task-set inertia) or other memory biases not explored here (e.g., inter-cue priming or context priming).

## Additional Files

The additional files for this article can be found as follows:

10.5334/joc.97.s1 Appendix A. List of Terminology.

10.5334/joc.97.s2 Appendix B. Model Description.

10.5334/joc.97.s3 Appendix C. Parameter Fitting and Sensitivity.

## Footnotes

Somewhat confusingly, this account is often referred to as the *proactive interference* account, as well.

Many conceptually-related models exist that model choice alone (e.g., recall), and are therefore less useful for our purposes. These other exemplar-based models should not be regarded as competitors, however, but rather family members.

Of course, regularities could be stored if, for instance, the perceiver consciously notices a regularity and resultantly encodes this conscious experience of the regularity. Though episodic modellers generally do not contest this notion (e.g., see a discussion by Hintzman, 1986), the modelling exercise is typically to see how far one can get with individual event memories alone.

Although mixing costs are sometimes discussed along with switch costs in the task switching literature (e.g., Meiran et al., 2008, assign a parameter to the mixing cost in their CARIS model to be discussed later), the timing mechanism used to simulate mixing costs in the PEP model is largely unrelated to the types of binding biases that we will discuss in the present manuscript.

It is also paramount for the stability of a learning system to weaken the impact of old information as it learns something new (Gerstner & Kistler, 2002). This is also true, for instance, with Hebbian learning, which is unstable without a decay (or learning rate) parameter (i.e., “what fires together, wires together” does not work long-term unless “what *stops* firing together, unwires”).

There is active debate as to whether the decreased influence of older events (“forgetting”) relative to newer ones is best conceptualized in terms of decay. As an alternative, older memories might not actually be “weakened,” but simply have less influence due to interference from events that followed them (e.g., see Oberauer & Lewandowsky, 2008). Implementation-wise, the PEP model does implement decay, though one might envision a different interference-based implementation. Practically speaking, this may make little difference for the simulation results themselves, as both solutions would result in diminished impact of older and older memories, but this remains an interesting theoretical consideration.

Instead of viewing feature integration effects as “confounds,” another view is that our model takes the vague concepts in cognitive control theories and specifies the “nuts and bolts” of control. As with many other concepts we discuss in this paper, this idea might depend on how one defines “cognitive control”. We will return to this point in the General Discussion.

Zero or (as in this case) close to zero errors is common in congruent trials in this type of neural network. When all information points to the same response, an error can only occur with an extremely “unlucky” string of random noise. In participants, congruent-trial errors can be similarly near non-existent in naming, but some small rate of errors is normal with, for instance, key press. The failure of the PEP (and related models) to produce more frequent congruent-trial errors does suggest an unmodelled source of error, perhaps due to guessing responses. This might be worth exploring further in future simulation work.

Note that we exclude parameterizations that are inherently inconsistent with theory and, indeed, nonsensical. For instance, a negative learning rate (i.e., where links between, say, a stimulus and response become *weaker* when they are paired together) obviously makes no sense. Parameters have certain logical possible ranges (see Table 2 for a list). For example, any proportion-change-type parameter (e.g., a learning rate) obviously must be between 0 and 1. It is further worth noting that allowing the model to violate these a priori ranges would not only be incoherent, but it would simply “break” the model entirely. For instance, a negative learning rate would prevent the model from continuing to respond correctly (and would not, by contrast, allow the model to fit reversed predictions).

As mentioned earlier, the switch cost is only a pure measure of reconfiguration with a process-pure task reconfiguration view, which is probably not a view endorsed by many, anyway.
