# Supplementary material for: An Episodic Model of Task Switching Effects: Erasing the Homunculus from Memory
Source: J Cogn. 2020 Sep 10;3(1):22. doi: 10.5334/joc.97 (PMC7485406; doi:10.5334/joc.97)
Supplement: Appendix A. — List of Terminology. [file joc-3-1-97-s1.pdf]

Appendix A: List of Terminology

Alternation: A feature (e.g., **task**) that changes from the previous trial.

Binding effects: Effects due to linkages between prior pairings of **stimuli**, **decisions**, **responses**, etc.

Complete alternation: When two elements of an event (e.g., **stimulus** and **response**) both change from the previous trial.

Complete repetition: When the pairing of two elements of an event (e.g., **stimulus** and **response**) repeats from the previous trial.

Cue: Information (e.g., colours) presented to participants on a trial to indicate the currently-relevant **task**.

Decision: A conceptual-level representation of a classification that is made about a stimulus (e.g., its parity, magnitude, or identity). Not to be confused with the **response**.

Episodic model: Another term for an **exemplar model**.

Exemplar model: A model of memory in which individual events are coded as discrete traces. This need not imply that the underlying code of the brain is localist (e.g., rather than distributed), but simply considers memory traces at a conceptual level.

Feature integration effects: Another term for **binding effects**.

Goal: A conceptual-level representation of the currently-relevant **task** to be performed (e.g., parity).

Partial repetition: When one element of an event repeats from the previous trial, but another element changes (e.g., same **stimulus**, but different **response**).

Proactive control: A form of sustained cognitive control recruited before stimulus onset. It has been discussed in conjunction with **task-set reconfiguration**. Not to be confused with **proactive interference**.

Proactive interference: Either another term for **task-set inertia** or a description of the finding that a previous **task-set** interferes with retrieval of a new one. Not to be confused with **proactive control**.

Reactive control: A late-correction control mechanism. Often used as another term for **task-set inertia**.

Stimulus: The target (or distracter) information presented (e.g., visually) to participants on a trial (e.g., digits).

Repetition: A feature (e.g., **task**) that repeats from the previous trial.

Response: The output that the participant makes (e.g., the key pressed). Not to be confused with the **decision**.

Switch cost: The observation that responding is impaired follow a change in the **task** relative to a repetition.

Task: The instructed set of rules that the participant needs to follow for the current trial (e.g., identify the parity of a digit).

Task-rule congruency: Whether the rules for two different **tasks** imply the same or different **responses** to the **stimulus**.

Task-set: Though often vague, can be defined as some collection of parameters or control settings that prepare the cognitive system identify the **stimuli** correctly, select and execute the **responses** according to instructions, etc.

Task-set inertia: The idea that the **task-set** is dynamically resolved in parallel to stimulus processing and is influenced by carryover activation of the prior **task-set** (or that resolution occurs involuntarily).

Task-set reconfiguration: The idea that the **task-set** must be "reprogrammed" as a discrete stage before preceding to stimulus processing (or that reprogramming occurs voluntarily).

Task switching paradigm: A paradigm in which participants must perform one **task** (e.g., parity decisions) on some trials and another task (e.g., magnitude decisions) on other trials, where both task repetitions and task alternations are possible.
