# Supplementary material for: An Episodic Model of Task Switching Effects: Erasing the Homunculus from Memory
Source: J Cogn. 2020 Sep 10;3(1):22. doi: 10.5334/joc.97 (PMC7485406; doi:10.5334/joc.97)
Supplement: Appendix B. — Model Description. [file joc-3-1-97-s2.pdf]

Appendix B: Model Description

Full, documented source code for this and all other versions of the PEP model are available for free download from the website of the lead author (leadserv.u-bourgogne.fr/~jschmidt/PEP/). Below, we explain the math of the model in full, along with explanations of the functioning of the model.

Activation function

As is standard in neural networks, on each processing cycle the *activation* of each node *i* is updated with Formula 1,

$$activation_i = activation_i(1 - decay) + input_i(decay) \qquad \textbf{(1)}$$

where $input_i$ is the stimulation received by the node (e.g., from connecting nodes), and *decay* is .086 for targets, cues, decisions, and responses, .007 for goals, and .0022 for episodes. As such, *activation* approaches *input* over time (e.g., with strong *input* the *activation* will gradually increase, whereas with weak or no *input* the *activation* will gradually decrease). *Input* is computed by first applying Formula 2,

$$retrieved_i = \frac{\sum_{j=1}^{n}\left(output_j(weight_{ji})(strength)\right)}{total} - .1 \qquad \textbf{(2)}$$

where $weight_{ji}$ is the connection strength between *j* and *i* and $output_j$ is the amount of activation sent (i.e., from the connected node *j*) via this weight. The *strength* value varies based on the information current sought. The normal "on" value is 1.14 for cues, 1.37 for goals, 3.4 for stimuli, 3.2 for decisions, and 3.5 for responses. These values are reduced to 15% for everything but cues and goals until the goal is determined (goal search), after which goal/cue search is reduced to 10% and the other nodes are set to their "on" values until a decision is determined (decision search), after which target search is decreased to 95% (response search). After a response, all *strength* values drop to 0, except for the response, which increases to 4.0 for 200 cycles (response coding) before also dropping to 0. The sum from Formula 2 is divided by the *total* summed retrieval for all nodes in the set (e.g., Response nodes), but only if *total* exceeds 1 (i.e., a limit on the amount of evidence retrievable per cycle). This is to produce proportional retrieval (Schmidt & De Houwer, 2016a). A small threshold of .1 is subtracted and the result of Formula 2 is then bounded between 0 (no *negative* retrieval) and 1 (technically impossible with the above formula). Note that this formula also produces congruency-based retrieval interference, and the lateral inhibition previously included in Decision nodes was resultantly discarded as unnecessary. This *retrieved* value is then input in Formula 3,

$$incoming_i = retrieved_i + noise \qquad \textbf{(3)}$$

where *noise* is computed from a Gaussian distribution (μ = .5, σ = .2, *range* = [0,1]), which is

multiplied by .012 for Input, .25 for Decision nodes, .66 for Goal nodes, and .0026 for Response nodes. No *noise* was added to Episode nodes. This noise merely adds some realistic variability in response times and accuracy. The result is then logistically transformed with Formula 4,

$$input_i = \frac{1}{1 + e^{-(incoming_i(8)-4)}} \quad \textbf{(4)}$$

This produces an S-shaped (aka, sigmoid) function that ranges between 0 and 1 and is fairly standard in neural networks (e.g., see Cohen, Dunbar, & McClelland, 1990).

Input nodes

Input nodes can be used to represent targets, distracters, or cues. The *input* activation to Input nodes (Formula 1) is additionally supplemented with a stimulus *bias* that is computed at the start of a trial with the Gaussian computation from the previous section multiplied by .8. On each cycle, *bias* adjusts toward a *signal* value with Formula 5,

$$bias_i = bias_i(1 - change) + signal_i(change) \quad \textbf{(5)}$$

where $signal_i$ is 1 for presented items and 0 for unpresented items and *change* is .034. Thus, *bias* approaches 1 over time for presented items, and 0 for unpresented items, but with some realistic initial noise in the inputs. This computation merely serves to produce human-like response time and error distributions. The stimulus *bias* itself, of course, is the stimulus-driven input that sets everything else in the model in motion. Output from Input nodes to Decision and Episode nodes (or between any two node types) is computed with Formula 6,

$$output_i = (activation_i - threshold)(weight_{ij}) \quad \textbf{(6)}$$

Where *threshold* is .5 for Input and Decision nodes, .15 for Episode nodes, .1 for goal nodes, and variable for Response nodes (see below).

Goal nodes

Goal nodes are activated exclusively via episodic retrieval, though presumably an adaptation would be possible for hardwired (e.g., non-arbitrary) cues. For Goal nodes, *incoming* activation (Formula 3) also includes lateral inhibition between other Goal nodes equalling 75% of the previous cycle activation state of the other goals. Goal nodes do not activate other nodes, but instead sensitize Decision nodes. In particular, Goal node activation is multiplied by the rate of .88 to connected Decision nodes. Decision node *preparedness* is computed by logistically transforming this input plus a baseline background preparedness of .4 using Formula 4, then adjusted across cycles using Formula 1 (decay rate = .1). See the following section for how *preparedness* influences evidence accrual.

Decision nodes

Decision nodes are activated via retrieval from episodes and any hardwired connections from Input nodes (*weight* = 6 for targets), both of which are jointly involved with the same *retrieved* formula described above (Formula 2). Decisions could presumably activate Response nodes via hardwired connections (i.e., with non-arbitrary response mappings), but these have not been implemented (the requisite code, however, is present in the model source code). Sensitivity of Decision nodes to input varies between 0 and 1, based on the *preparedness* mentioned above.

Response nodes

Response nodes receive input exclusively via episodic retrieval (i.e., unless non-arbitrary decision-response mappings are programmed). The model responds when one of these nodes exceeds the response *threshold*, which can vary between .25 and .45 with the temporal expectancy mechanism (see below).

Episode nodes

Several features of Episode nodes change in Version 3.0 of the model. A major change relates to instruction encoding. With the partial exception of trial-and-error learning research (Thorndike, 1911), instructions tell participants which keys to press to which stimuli or even which tasks to do when presented with different task cues (i.e., which goals to prioritize). Instructed memories were coded directly, each of which could encode cue-goal links or decision-response links with weights between the instructed nodes of 1.

Though this is generally enough for the model to maintain instructions, it is possible that instructions will be “lost” in the face of a bad streak of errors. This, of course, is psychologically realistic, as impairments are observed following errors (e.g., Nuttin & Greenwald, 1968), including the repetition of specific errors (see Marx, 1971; Marx & Marx, 1980; Marx, Witter, & Farbry, 1973; for a review, see Koppenaal, 1960). Although a full treatment of post-error correction is far beyond the scope of this article (see Holroyd, Yeung, Coles, & Cohen, 2005; Ohlsson, 1996), we did add some code to prevent catastrophic loss of instructions. In particular, when an error was made, the model searched for instructions matching the identified cue and selected decision (assuming there was one) and re-strengthened the weights by adding .285 to the current weight of the instruction memory. Note that we re-strengthen the original instruction memory only for computational simplicity. The conceptual idea, however, is that when a participant makes an error they (perhaps only probabilistically) try to “remind” themselves of the correct instruction and re-encode it. If not for practical coding concerns (e.g., throwing off a loop index variable), this would have been encoded as a new episode. Other than keeping the model responding with reasonable accuracy for all simulated participants, this code plays no key role in any of the results in this paper.

Experienced-based episodes were encoded dynamically, but otherwise function

identically to their instruction-based counterparts. Importantly, all other nodes in the model (Input, Goal, Decision, and Response) wire to each newly created episode with one formula, computed after each processing cycle. In particular, node *j* is recorded into episode *i* using Formula 7,

$$weight_{ij} = weight_{ij}(1 - write) + incoming_i(write) \qquad \mathbf{(7)}$$

where *write* is .01 and $incoming_i$ is the activation received from node *j*. Importantly, $weight_{ij}$ no longer changes if *incoming* is smaller than the current *weight*. This was deemed important, because, for instance, a stimulus strongly active early on that subsequently decays (e.g., cues) would get "un-encoded" without this consideration. The *incoming* value is computed with Formula 8,

$$incoming_i = search_j\left(activation_j - threshold_j\right) \qquad \mathbf{(8)}$$

the values for which were defined above. During the experiment, new Episode nodes started the trial with weights of −.11. This was not required for the model to fit the current data, but was added simply because it is more comparable to most other exemplar-based models where mismatching features count "against" retrieval of the memory. Weights are diminished by a rate of .02 if an error is made. The newly-created episode also receives persistent activation of .75 until the end of the trial.

Note that activated Episode nodes send output activation (but never negative) to *all* connected nodes (Input, Goal, Decision, and Response) via the exact same connection *weight* that the respective nodes send activation. When Episode nodes are activated during a trial, the connection *weights* are reduced at the end of the trial using Formula 9,

$$weight_{ij} = weight_{ij}\left(1 - peak_i(loss)\right) \qquad \mathbf{(9)}$$

where $peak_i$ is peak episode *activation* and *loss* is .045. For discussions of the importance of this loss for the stability and high learning rate of a memory model see Gerstner and Kistler (2002) and Schmidt and colleagues (2016).

Though not relevant for the present paper, response times are also encoded in episodes and used to anticipate when to respond (Schmidt & Weissman, 2016; Schmidt, 2013a, 2016a). The response time connection weight starts at .02 and decays in the same way as other weights. The threshold for Response nodes is determined by current *pace* (i.e., expected time to respond), which is set at the start of the trial with Formula 10,

$$pace = pace(rate) + rt(1 - rate) \qquad \mathbf{(10)}$$

where *rate* is .5 and *rt* is the response time on the immediately preceding trial. Thus, *pace* is codetermined by the just-made response and previous pace. During the trial, *pace* adjusts on each cycle via episodic retrieval with Formula 11,

$$pace = \sum_{i=1}^{n-1} (rt_i - pace)^2 (weight_i)(activation_i - threshold) \qquad \mathbf{(11)}$$

where *i* is the episode number, *n* is the current trial number, rt_i is the stored response time for episode *i*, $activation_i$ is the activation of episode *i*, and *threshold* is the threshold for episodic retrieval. Note that this formula is only computed for episodes more active than the threshold. The result can be a speeding or slowing of *pace* depending on what is retrieved (see Schmidt, 2016a). The response threshold is then determined with Formula 12,

$$threshold = .25 + \frac{(cycle - pace)^2}{50{,}000} \qquad \mathbf{(12)}$$

where the result of the formula is restricted between .25 and .45. As such, the baseline response *threshold* is .45, but the threshold decreases as the absolute difference between *cycle* and *pace* decreases (i.e., as time approaches the expected time to respond).

References

1. Cohen, J. D., Dunbar, K., & McClelland, J. L. (1990). On the control of automatic processes: A parallel distributed-processing account of the Stroop effect. *Psychological Review, 97,* 332–361.
2. Holroyd, C. B., Yeung, N., Coles, M. G. H., & Cohen, J. D. (2005). A mechanism for error detection in speeded response time tasks. *Journal of Experimental Psychology: General*, *134*, 163–191.
3. Koppenaal, R. J. (1960). Repetition of errors in human multiple-choice learning. *Psychological Reports*, *7*, 269–286.
4. Marx, M. H. (1971). Increased probability of error repetition as a function of number of successive prior repetitions. *Perceptual and Motor Skills*, *32,* 544–546.
5. Marx, M. H., & Marx, K. (1980). Confirmation of the stubborn-error effect in human multiple-choice verbal-learning. *Bulletin of the Psychonomic Society*, *16*, 477–479.
6. Marx, M. H., Witter, D. W., & Farbry, J. (1973). Greater repetition of errors under performance compared to observation in multiple-choice human learning. *Perceptual and Motor Skills*, *37*, 949–950.
7. Nuttin, J., & Greenwald, A. G. (1968). *Reward and punishment in human learning: Elements of a behavior theory*. New York: Academic Press.
8. Ohlsson, S. (1996). Learning from performance errors. *Psychological Review, 103,* 241–262.
9. Thorndike, E. L. (1911). Animal intelligence. New York: MacMillan.
