# Supplementary material for: An Episodic Model of Task Switching Effects: Erasing the Homunculus from Memory
Source: J Cogn. 2020 Sep 10;3(1):22. doi: 10.5334/joc.97 (PMC7485406; doi:10.5334/joc.97)
Supplement: Appendix C. — Parameter Fitting and Sensitivity. [file joc-3-1-97-s3.pdf]

Appendix C: Parameter Fitting and Sensitivity

We were initially able to produce reasonable qualitative fits to initial simulations (along with several other simulations not included here) with one fixed parameter set (i.e., using intuitive parameter adjustment). However, we followed this with quantitative fits using an evolutionary algorithm (for a review, see Vikhar, 2016). In particular, initial parameters were seeded and a population of “parent” samples were run. Each parent used the seeded parameters, except that each parameter had a small chance to “mutate” (i.e., change by some Gaussian-distributed amount). Then, many generations of “child” simulations were run. Each child was “bred” from a random subset of three neighboring parents, with each parameter randomly selected from one of these parents, again with a small chance of mutation. For each run, error was computed by determining how well the cycle times and simulated errors matched the participant sample means. This “match” was the summed squared error (*SSE*) of all response time and error rate cells. Because error rates are (naturally) much smaller numbers than response times, squared error for error rate cells was multiplied by 20 (which still weighs response times more heavily, but a little less dramatically so). Overall longer participant response times were allowed with a nondecision time adjustment (constrained to be ≥ 0 and fit to 143). In particular, the difference between mean participant response times and mean model cycle times (if positive) was added to each cycle time for each cell. Each child with greater error than the worst (i.e., highest error) parent in the “neighborhood” was “murdered” (i.e., not retained in the population). A child that outperformed the worst parent “murdered” said parent (i.e., replacing it in the population). The rate and degree of mutations can be adjusted in the Evolve.java class, and additional control parameters are also available (though were not used). As mentioned in text, the same approach was used to try to fit the model to “reversed” predictions in the parameter sensitivity analysis.

Although the full Java source code is available for download, below we present Java pseudocode to illustrate the basic function of the final evolutionary algorithm:

```
for(parent=0; parent<number of parents; parent++){
  for(trait=0; trait<number of traits; trait++){
    random chance to mutate parent trait by a random amount;
  }
}
run each parent in PEP model;
find parent with worst error;
for(child=0; child<number of children; child++){
  set neighbourhood of nearby parents to breed;
```

```
  for(trait=0; trait<number of traits; trait++){
    randomly select trait from parents;
    random chance to mutate child trait by a random amount;
  }
  run child in PEP model;
  if child error is less than worst parent error{
    kill worst parent, replace with child;
    find new worst parent;
  }
  else kill child, retain parent;
}
```
